# Diversion of Arginine’s dietary metabolic fate in proteinuric kidney disease

**DOI:** 10.21203/rs.3.rs-9927381/v1

**Published:** 2026-06-17

**Authors:** Maria Chrysopoulou, Xavier de la Rosa, Yifan Tan, Elina Kovalenko, Oliver Hahn, Jesper Frank Andersen, Moritz Lassé, Vladimir Matchkov, Vera Christine Wulfmeyer, Johannes Jaegers, Anja M. Billing, Elizabeth Colby, Markus Bleich, Bernhard Schermer, Sarantos Kostidis, Martin Giera, Janos Peti-Peterdi, Eugene P. Rhee, Morgan E. Grams, Sushrut S. Waikar, Meryl Waldman, Nicola M. Tomas, Moin A Saleem, Nina Himmerkus, Insa M. Schmidt, Peder Berg, Fatih Demir, Markus M. Rinschen

**Affiliations:** 1 Department of Biomedicine, Aarhus University, Aarhus, Denmark; 2 III. Department of Medicine, University Medical Center Hamburg-Eppendorf, Hamburg, Germany; Hamburg Center for Kidney Health, University Medical Center Hamburg-Eppendorf, Hamburg, Germany; 3 Bristol Renal, University of Bristol, Bristol, United Kingdom; 4 Institute of Physiology, University of Kiel, Kiel, Germany; 5 Cologne Excellence Cluster on Cellular Stress Responses in Aging-Associated Diseases (CECAD), Faculty of Medicine and University Hospital Cologne, University of Cologne, 50931 Cologne, Germany, and Department II of Internal Medicine and Center for Molecular Medicine Cologne, Faculty of Medicine and University Hospital Cologne, University of Cologne, 50931 Cologne, Germany.; 6 Center of Proteomics and Metabolomics, Leiden University Medical Center, Leiden, The Netherlands.; 7 Division of Nephrology and Hypertension, Department of Medicine, Keck School of Medicine, University of Southern California, Los Angeles, CA 90033, USA; Department of Physiology and Neuroscience, Zilkha Neurogenetic Institute, Keck School of Medicine, University of Southern California, Los Angeles, CA 90033, USA.; 8 Division of Nephrology, Department of Medicine, Massachusetts General Hospital, Boston, MA, USA; 9 Department of Medicine, New York University Langone School of Medicine, New York, NY, USA; 10 Section of Nephrology, Department of Medicine, Boston University Chobanian & Avedisian School of Medicine and Boston Medical Center, Boston, MA, USA; 11 NIDDK, NIH, Bethesda, MD, USA

## Abstract

Proteinuria predicts chronic kidney disease progression and cardiometabolic mortality, yet how sustained urinary protein loss reshapes interorgan metabolism remains unclear. Here, we tested whether proteinuric kidney disease alters whole-body amino-acid and nitrogen handling by applying long-term dietary arginine isotope tracing in a proteinuric podocin-mutant mouse model, complemented by *ex vivo* nephron-segment metabolism and human cohort analyses. Because arginine connects amino acid use in protein synthesis, amino acid catabolism, and the urea cycle, the dietary isotope label allowed us to follow both metabolite flux and protein incorporation across organs over time. Proteinuria did not trigger a broad compensatory increase in protein synthesis. Instead, it caused a kidney-centered rerouting of arginine metabolism. Arginine use shifted away from hepatic ureagenesis and toward renal glutamate-, proline-, and aspartate-linked fate. Across organs, most proteins incorporated recycled arginine, whereas albumin preferentially incorporated diet-derived arginine, identifying albumin as a major non-recycled arginine sink during proteinuric disease. In the kidney, proximal nephron segments showed coordinated remodeling of arginine and proline metabolism in association with tubular albumin handling. This rerouting favored proline synthesis and early collagen incorporation before overt fibrosis was established. Isotope-resolved nitrogen tracing indicated that this renal diversion of arginine occurs at the expense of coordinated nitrogen and acid handling, as reflected by reduced urinary ammonium excretion, tissue accumulation of arginine-derived ammonia, and altered abundance of enzymes involved in ammonium detoxification. In humans, circulating aspartate was elevated during active proteinuric disease and decreased in remission, independently of glomerular filtration rate, and both aspartate and proline associated with histological markers of fibrotic remodeling in biopsies from glomerular disease patients. Consequently, in proteinuric mice, a high dietary content of aspartate/asparagine further aggravated collagen accumulation and fibrosis-related remodeling of high-proline-content proteins, while impairing acid excretion. Together, these findings define proteinuric kidney disease as a chronic interorgan nitrogen-redistribution state that links selective protein turnover, impaired ammonium disposal, acid retention, and a renal profibrotic metabolic environment.

## Introduction

With 800 million people affected worldwide, kidney disease represents a major global health burden^1^. Proteinuria is a defining feature of kidney disease, particularly in nephrotic syndrome and other glomerular diseases, where it reflects failure of the glomerular filtration barrier^2^. Beyond serving as a marker of renal injury, sustained urinary protein loss accelerates chronic kidney disease progression and contributes independently to systemic complications, including cardiovascular disease^3^. Severe proteinuria is characterized by urinary loss of circulating albumin and consequent hypoalbuminemia, which contribute to hyperlipidemia and, in part, hypercoagulability^4–6^. However, targeting hyperlipidemia or hypercoagulability has not emerged as a clear strategy to improve the prognosis of severe proteinuria^3,6–8^. Thus, the molecular and metabolic adaptations that connect glomerular protein loss to progressive kidney injury and interorgan dysfunction remain incompletely understood.

Whether sustained urinary protein loss rewires whole-body amino-acid fate, and whether this metabolic redistribution contributes directly to tubular injury and fibrotic remodeling, remains unknown. Amino-acid metabolism represents a plausible mechanistic link between proteinuria, renal tubular stress, and systemic metabolic adaptation^9^. Constant urinary protein loss and hypoalbuminemia have been proposed to increase compensatory protein synthesis, supported by tracer incorporation into the plasma proteome^10,11^, whereas proxy analyses suggest no major alteration in global protein turnover^12^. However, in proteinuric disease, nitrogen disposal and amino-acid incorporation into individual proteins and organs have not been resolved.

Arginine is well suited to interrogate this question because it links dietary amino-acid utilization, protein incorporation, ureagenesis, nitrogen disposal, nitric oxide metabolism, and proline biosynthesis. In addition to being incorporated into newly synthesized proteins, arginine is a central intermediate in the urea cycle, where nitrogen derived from free ammonium and aspartate is incorporated into urea for excretion^13^. From a physiological perspective, ammonium detoxification and nitrogen disposal require close kidney–liver cooperation: the liver converts ammonium into urea, whereas the kidney excretes urea and ammonium and contributes to systemic glutamine and nitrogen handling. Together, these processes maintain nitrogen homeostasis and acid–base balance. Arginine also participates in pathways directly relevant to kidney disease, including nitric oxide production, polyamine synthesis, and the ornithine–glutamate–proline axis, which supports collagen production and fibrotic remodeling.

Comprehensive analysis of these physiological pathways remains constrained by methodologies that do not capture key aspects of system-level physiology, including both protein turnover and the metabolic fate of their building blocks. Label-free metabolomics and proteomics provide static snapshots of metabolic states without identifying the source of the detected signals. Also, tracer analyses performed by infusion are limited by short labeling durations, which prevent the assessment of protein turnover^12^, and fail to account for the active metabolism of dietary compounds along the digestive tract, both critical layers of physiological processes and regulation. Finally, methods for ammonium detection compatible with mass spectrometry have only recently become available^14^.

Here, we hypothesized that proteinuria disrupts coordinated interorgan metabolism and perturbs the metabolic communication between organs. We overcame the two issues listed above by pioneering dietary isotope labeling, coupled with metabolomics and proteomics analyses, applied to a late-onset, proteinuric podocin-mutant mouse model (CRISPR NPHS2^R231Q/A286V^), that resembles human disease physiology^15^. Using these approaches, together with *ex vivo* nephron-segment tracing and human cohort analyses, we identified a kidney-centered network of interorgan metabolic communication invoked by proteinuria. Proteinuric kidney disease redirected dietary arginine utilization toward renal glutamate-, proline-, and aspartate-linked metabolism, and linked impaired ammonium disposal and acid handling with a profibrotic metabolic environment in the kidney.

## Results

### Proteinuria induces kidney-dominant proteomic remodeling of nitrogen-handling and metabolic pathways

**Figure 1.**
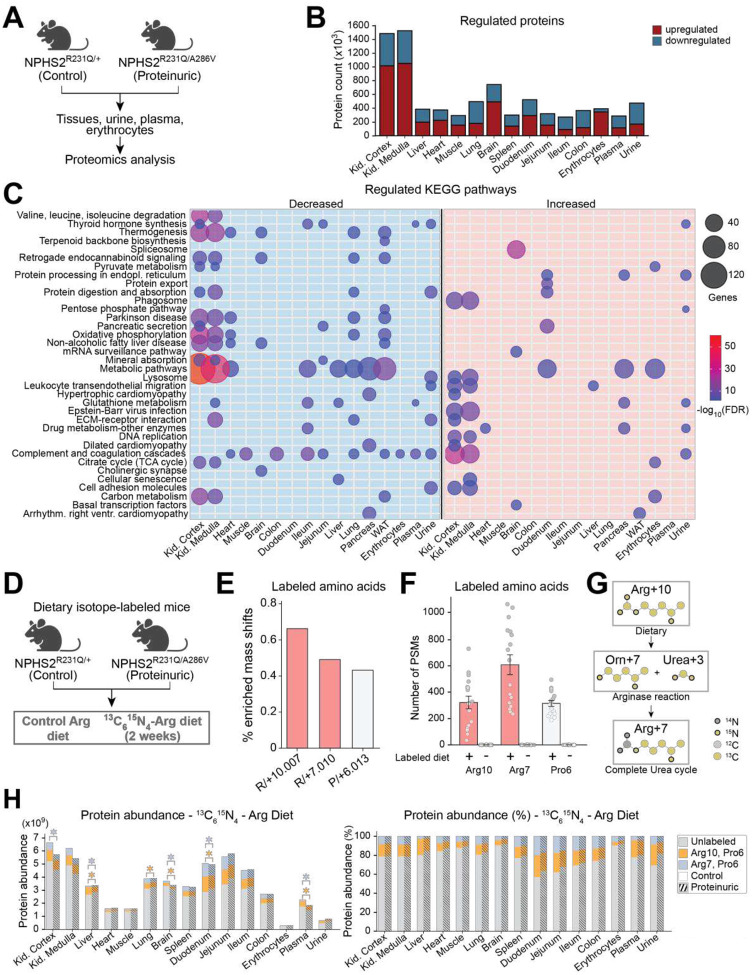
Multi-organ proteomics of proteinuric kidney disease mice identifies kidney-centered remodeling and incorporation of recycled arginine into the proteome. **A.** Study design of sample collection and processing from control (NPHS2^R231Q/+^, n=6) and proteinuric (NPHS2^R231Q/A286V^, n=4) mice. **B.** System-wide differential protein expression between control and proteinuric mice. Statistical analysis was performed using *limma*. Differentially expressed proteins: log_2_FC > |0.3| and P value < 0.05. **C.** System-wide KEGG pathway enrichment analysis of differentially expressed proteins between control and proteinuric mice. **D.** Study design of mice fed a control (n=9) and isotope-labeled arginine (^13^C_6_^15^N_4_) (n=9) diet. **E.** Enrichment of the top three isotope-labeled amino acids and their corresponding mass shifts as identified by the untargeted PTM search strategy in FragPipe (open search analysis). **F.** Quantification of peptide spectrum matches (PSMs) detected by open search in mice fed the ^13^C_6_^15^N_4_-arginine diet; points represent individual samples. **G.** Schematic of isotope-tracing of dietary arginine through the arginase reaction and urea cycle. **H.** System-wide absolute (left) and percent (right) protein abundance after incorporation of Arg10/Pro6 and Arg7/Pro6 in mice fed the ^13^C_6_^15^N_4_-arginine diet.

We utilized a previously characterized genetic model of proteinuric kidney disease, whose renal phenotype was confirmed^15^. Compound heterozygous NPHS2^R231Q/A286V^, mimicking one of the most frequent genetic causes for late onset proteinuric kidney disease^15,16^, served as proteinuric kidney disease mice, and non-proteinuric NPHS2^R231Q/+^ littermate mice were used as control. To define organ-resolved protein changes in proteinuria, we performed quantitative proteomics across 13 organs as well as plasma, urine, and erythrocytes from proteinuric young adult NPHS2^R231Q/A286V^ mice and non-proteinuric NPHS2^R231Q/+^ controls (Fig. 1A). Mice at 10 weeks of age show no significant interstitial fibrosis^15^. Notably, inter-organ proteome comparisons revealed that the kidney, both cortex and medulla, exhibited the strongest proteomic remodeling. The kidney cortex and medulla contained the highest numbers of regulated proteins among tissues (Fig. 1B, **Suppl. Table 1**), consistent with the kidney being the primary affected organ. Kyoto Encyclopedia of Genes and Genomes (KEGG) enrichment analysis highlighted pathway-level remodeling predominantly occurring in the kidney. Downregulated proteins were enriched for metabolic pathways, including amino acid and energy metabolism, while upregulated proteins were enriched for processes consistent with altered protein handling and immune responses (Fig. 1C, **Suppl. Table 2**).

### Dietary arginine incorporation into proteins is not globally increased in proteinuria

Because kidney-dominant remodeling involved nitrogen-related metabolism and protein-processing pathways, we next asked whether proteinuria triggers a compensatory increase in protein synthesis or instead alters the metabolic fate of dietary amino acids. We selected dietary arginine isotope labeling because arginine reports both incorporation into newly synthesized proteins and routing through nitrogen-disposal and proline-generating pathways^13^. Mice were fed a diet containing stable isotope-labeled arginine (^13^C_6_/^15^N_4_), which generates a +10.007 Da mass shift (Arg10) when diet-derived arginine is incorporated directly into newly synthesized proteins (Fig. 1D). Unbiased open-search analysis confirmed robust incorporation of dietary +10 arginine label and further identified enrichment of +7 arginine and +6 proline labeled states (Arg +7.010 Da; ^13^C_5_^15^N_2_; [Arg7] and Pro +6.013 Da; ^13^C_5_^15^N_1_ [Pro6]) (Fig. 1E, **Suppl. Table 3**). These signals were absent when no isotopes were fed (Fig. 1F). These signals reflect metabolic reprocessing of dietary arginine before protein incorporation: Arg7 is generated after loss of the labeled urea moiety during urea-cycle passage, whereas Pro6 reflects conversion of arginine through the ornithine–glutamate–proline axis (Fig. 1G). Thus, the dietary arginine label allowed us to distinguish direct incorporation of diet-derived arginine from incorporation of metabolically recycled arginine-derived amino-acid pools.

We then performed multi-organ proteomics profiling analysis, considering two arginine labeling states (Arg10/Pro6; Arg7/Pro6) consistent with previous in vivo stable isotope labeling analyses for proteomics^17–19^. In the isotope-fed animals, the fractional contribution of newly synthesized (labeled) proteins did not differ between proteinuric and control mice in most tissues (Fig. 1H, **Suppl. Table 4**). Brain, duodenum, liver, plasma, and partially the kidney and lung showed reduced labeled protein intensities in proteinuric mice (**Suppl. Table 4**). In the absence of isotope feeding, incorporation of Arg10/Pro6 or Arg7/Pro6 was not detectable (Suppl. Fig. 1A, **Suppl. Table 4**). To test whether changes in protein abundance were driven by altered synthesis, per-protein incorporation ratios were compared with differential protein abundance. There was a slight, and for the Arg7/Pro6 mass shifts, significant negative correlation between isotope incorporation and abundance changes in plasma proteins (Suppl. Fig. 1B, C, **Suppl. Table 5**). Here, reductions in protein abundance through proteinuria were associated with greater changes in isotope incorporation rate (Suppl. Fig. 1B, C), consistent with higher protein synthesis of proteins depleted under proteinuric conditions (Suppl. Fig. 1D, E, **Suppl. Table 6**). This relationship was absent in other tissues, where protein synthesis appeared uncoupled from protein abundance (Suppl Fig. 1F, **Suppl. Table 7**).

Because albumin is the dominant circulating protein and the major urinary protein lost during proteinuria, we next examined isotope-labeled amino acid incorporation specifically in albumin peptides. In contrast to the broader proteome-wide labeling pattern, albumin showed preferential incorporation of Arg10/Pro6, indicating that albumin synthesis relies strongly on directly diet-derived arginine rather than metabolically recycled arginine-derived pools (Arg7/Pro6) (Suppl. Fig. 1G, **Suppl. Table 8**). An alternative, targeted bioinformatic analysis of albumin peptides in liver, the site of albumin synthesis, confirmed predominant Arg10 labeling, supporting albumin as a selective sink for non-recycled dietary arginine during proteinuric disease (Suppl. Fig. 1H, **Suppl. Table 9**). Thus, whereas most proteins incorporated substantial recycled arginine-derived labeling signatures (Suppl. Fig. 1H), albumin preferentially used the direct dietary arginine pool.

### Proteinuria shifts dietary arginine labeling from hepatic ureagenesis toward renal arginine-derived metabolism

**Figure 2.**
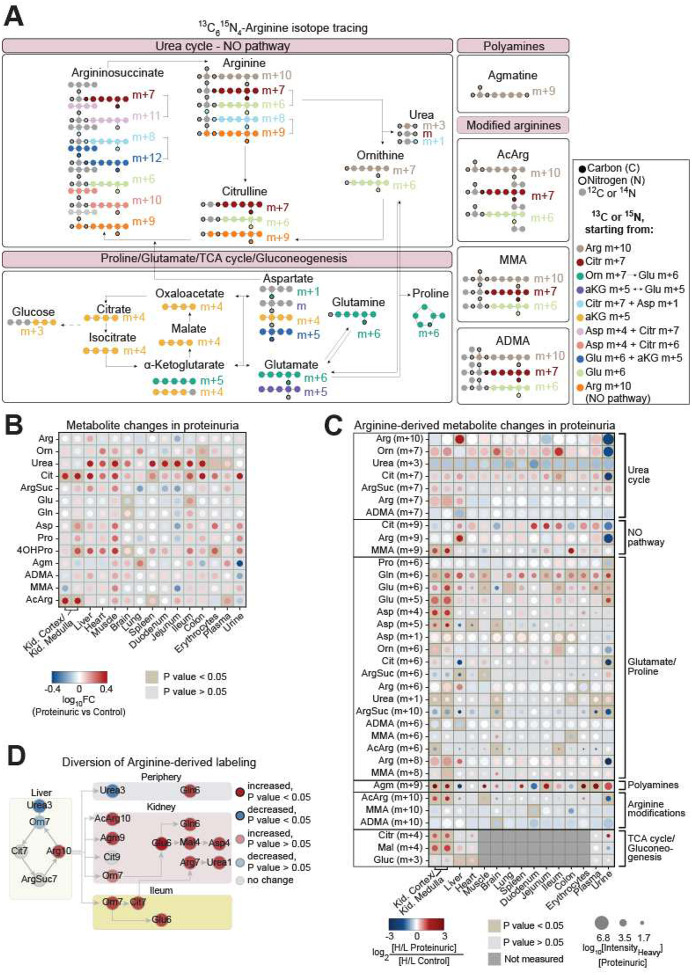
System-wide dietary arginine isotope tracing reveals a diversion of systemic arginine fate from the liver towards the kidney. **A.** Schematic representation of ^13^C_6_^15^N_4_-arginine tracing across arginine-related metabolic pathways. **B.** System-wide differential abundance of arginine-related metabolites between control and proteinuric mice receiving the ^13^C_6_^15^N_4_-arginine diet. Statistical significance was assessed using a two-sided Mann–Whitney U test (P value < 0.05). **C.** System-wide differential abundance of arginine-derived isotope-labeled metabolites between control and proteinuric mice. Statistical significance was assessed using a two-sided Mann–Whitney U test (P value < 0.05). **D.** Schematic summary of the major labeled metabolites detected in liver, kidney, ileum, and peripheral tissues based on the isotope-tracing analysis shown in C. Blue circles: significantly decreased, red circles: significantly increased, grey circles: unchanged metabolite abundance.

We next mapped inter-organ arginine metabolism using targeted metabolomics across organs, plasma, urine, and erythrocytes. The traced network covered urea-cycle intermediates, polyamine precursors, nitric oxide–related metabolites, and the ornithine–glutamate–proline interconversion (Fig. 2A). Under control-diet conditions (unlabeled), arginine-related metabolite abundances were broadly altered in proteinuria (Fig. 2B, **Suppl. Table 10**). Urea, citrulline, and 4-hydroxyproline increased systemically, while the kidney additionally showed increased aspartate and acetyl-arginine. Brain glutamate decreased with an increase in glutamine, and the ileum showed increased glutamate (Fig. 2B).

Stable-isotope readouts, expressed as log_2_ ratios of labeled to unlabeled metabolite isotopologues, revealed organ-specific changes in the fate of diet-derived arginine (Fig. 2C, **Suppl. Table 11**). In the liver, m+10 arginine labeling increased, whereas m+3 urea and m+7 ornithine labeling decreased, consistent with reduced commitment of diet-derived arginine to hepatic ureagenesis. In the ileum, increased m+7 ornithine and citrulline labeling suggested enhanced intestinal processing of dietary arginine. In the kidney, selective enrichment of m+7 arginine, together with downstream labeling patterns in glutamate-, proline-, and aspartate-linked metabolites, indicated increased renal utilization of reprocessed arginine-derived pools. Together, these isotope-labeling patterns support a model in which proteinuria shifts dietary arginine fate away from hepatic ureagenic labeling and toward renal arginine-derived metabolism (Fig. 2D).

### Proximal nephron segments are reprogrammed in proteinuria toward glutamate/proline production through albumin-associated metabolic enzyme changes.

The kidney-enriched arginine-derived isotope signatures raised the question of which nephron compartments can generate these metabolites and whether this capacity is altered by proteinuria. Several large-scale and protein-detection methods have localized urea cycle enzymes in the kidney^20,21^, with only indirect readouts of enzymatic function.

**Figure 3.**
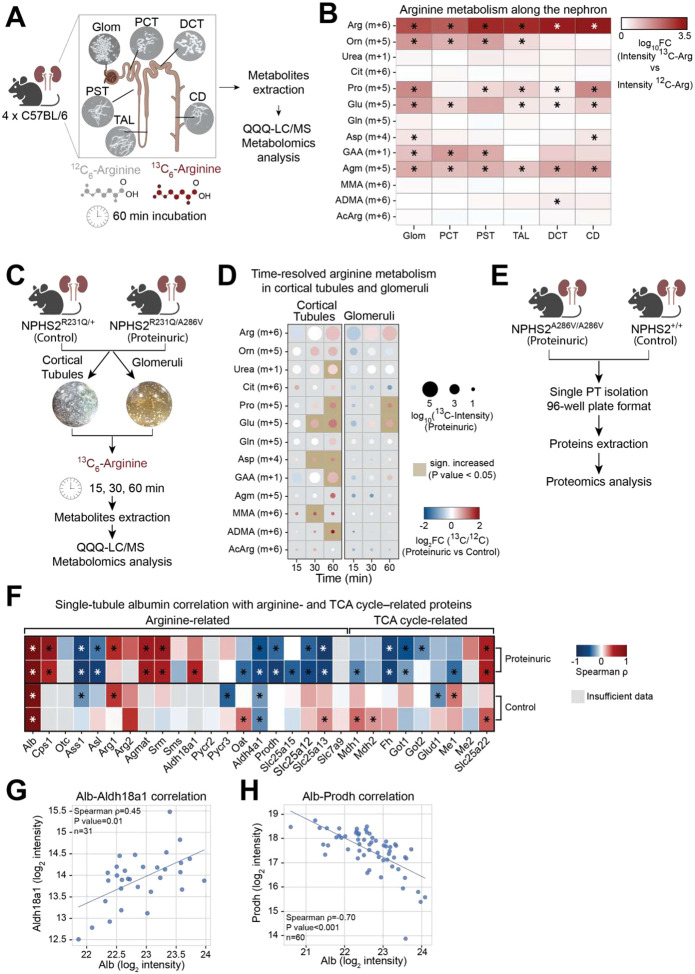
Proteinuria redirects arginine metabolism toward glutamate and proline synthesis in nephron segments. **A.** Experimental workflow for analysis of arginine metabolism in isolated nephron segments. **B.** Abundance of isotope-labeled arginine-related metabolites in isolated nephron segments. Statistical significance was assessed using a two-sided Mann-Whitney U test (P value < 0.05). **C.** Experimental workflow for analysis of time-resolved arginine metabolism in cortical tubules and glomeruli. **D.** Differential abundance of isotope-labeled arginine-related metabolites in cortical tubules and glomeruli derived from control and proteinuric mice after 15, 30, and 60 minutes of incubation. Statistical significance was assessed using a two-sided Mann-Whitney U test (P value < 0.05). **E.** Experimental workflow for proteomic analysis of single-tubule data from control and proteinuric mice. **F.** Spearman correlation of albumin with a selected subset of arginine-related and TCA cycle-related proteins from single-tubule data in control and proteinuric mice. **G.** Representative intra-mouse Spearman correlation of albumin with Aldh18a1, a protein central for proline synthesis. Each dot denotes the protein abundance of one proximal tubule. **H.** Representative intra-mouse Spearman correlation of albumin with Prodh, a protein degrading proline. Each dot denotes the protein abundance of one proximal tubule.

To localize renal arginine metabolism, we first performed *ex vivo* isotope tracing in microdissected nephron segments from wild-type mice. This approach preserves segment-specific metabolic activity under controlled oxygenated conditions and is well-suited to assess intrinsic renal metabolic capacity^22–26^. Microdissected nephron segments were incubated with ^13^C_6_-arginine (Arg+6), and isotope-labeled arginine-derived metabolites were quantified over time (Fig. 3A). Across segments, labeled arginine increased as expected. Labeled ornithine was generated in glomeruli, proximal convoluted tubules, proximal straight tubules, and the thick ascending limb, whereas labeled proline, glutamate, and agmatine were detected broadly along the nephron. By contrast, labeled guanidinoacetate was restricted to glomeruli and proximal tubule segments (Fig. 3B, **Suppl. Table 12**). These data demonstrate broad but segment-localized capacity for arginine metabolism along the nephron, with proximal segments showing particularly diverse arginine-derived products previously found *in vivo* (Fig. 2D).

We next asked whether this intrinsic renal arginine-metabolizing capacity was altered by proteinuria. Cortical tubules and glomeruli were isolated from proteinuric NPHS2^R231Q/A286V^ mice and littermate controls and incubated *ex vivo* with labeled arginine (Fig. 3C). This sample contains >80% proximal tubules^27^. Compared to control tubules, proteinuric cortical tubules showed increased formation or accumulation of labeled urea, glutamate, proline, and guanidinoacetate over time, indicating enhanced arginine-derived nitrogen and carbon processing in the tubular compartment. By contrast, proteinuric glomeruli showed slower overall labeling dynamics, with delayed, yet significant increases in labeled glutamate and proline (Fig. 3D, **Suppl. Table 13**). Thus, proteinuria selectively enhanced arginine-derived metabolic activity in cortical tubules.

To link tubular albumin handling with metabolic remodeling, we performed single-tubule proteomics in control and proteinuric podocin-mutant mice as previously described^28^. In total, 61 and 57 dissected S1 proximal tubules were collected from two proteinuric mice, and 87 and 95 tubules from two control mice, resulting in an average of 2151.2 ± 31.7 detected proteins per tubule (mean ± SEM, n = 300 tubules; Suppl. Fig. 2A, **Suppl. Table 14**). Principal component analysis clearly separated proteinuric from non-proteinuric tubules (Suppl. Fig. 2B, **Suppl. Table 15**). We then used albumin abundance across individual tubules as a marker of tubular protein reabsorption burden and examined its relationship with enzymes involved in arginine metabolism and the TCA cycle (Fig. 3E). Many arginine- and TCA-cycle-related proteins showed stronger negative correlations with albumin abundance in proteinuric tubules than in controls, consistent with broad metabolic suppression in albumin-loaded tubules. However, selected enzymes showed the opposite pattern. ALDH18A1, which supports proline synthesis, and CPS1, which supports ammonium fixation, correlated positively with albumin abundance (Fig. 3F–G, Suppl. Fig. 2C–D, **Suppl. Table 16**). Conversely, PRODH, which catalyzes proline catabolism, correlated negatively with albumin abundance in both proteinuric mice (ρ = −0.70, p < 0.001, n = 60/61; ρ = −0.41, p = 0.002, n = 56/57; Fig. 3H, Suppl. Fig. 2E). Thus, albumin-exposed proteinuric tubules showed a selective remodeling pattern that suppresses proline catabolism while favoring proline synthesis and altered nitrogen handling.

### Proline incorporation into proteins is an arginine sink in proteinuria.

**Figure 4.**
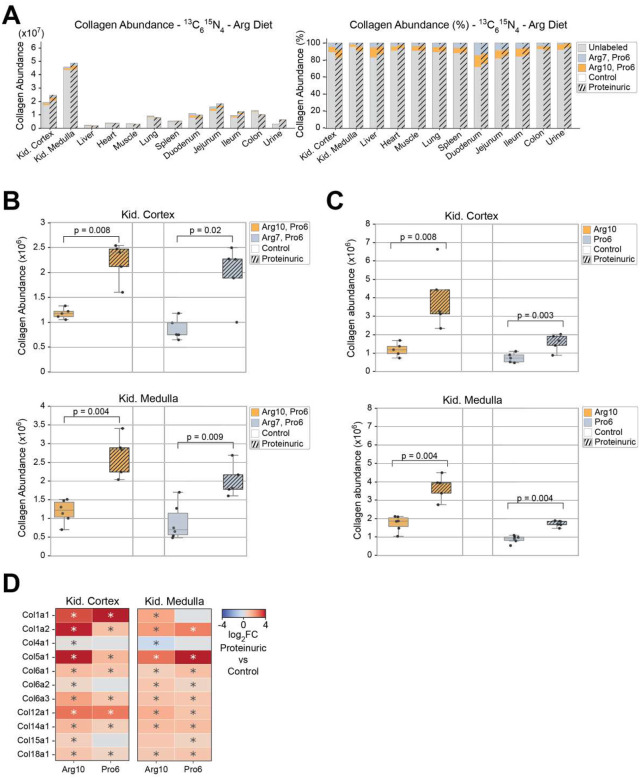
Incorporation of arginine-derived proline into proteins represents a metabolic sink for arginine in proteinuria. **A.** System-wide absolute (left) and percent (right) collagen abundance after incorporation of Arg10/Pro6 and Arg7/Pro6 in mice fed the ^13^C_6_^15^N_4_-arginine diet. **B-C.** Per-mouse summed collagen intensities reflecting incorporation of labeled amino acids in control and proteinuric mice. Each dot represents one mouse. Boxes indicate the median and interquartile range (IQR), and whiskers extend to 1.5 × IQR. P values were calculated using a two-sided Mann–Whitney U test. Panel B shows collagen intensities derived from peptides incorporating Arg10/Pro6 and Arg7/Pro6, whereas Panel C shows collagen intensities derived from peptides incorporating Arg10 and Pro6. **D.** Differential abundance of collagen proteins enriched in labeled amino acids (Arg10, Pro6) in kidney cortex and medulla between control and proteinuric mice.

The nephron-segment tracing data indicated enhanced arginine-derived proline generation in proteinuric tubules, yet steady-state proline labeling was not increased in whole-kidney metabolite pools (Fig. 2C). We therefore asked whether newly generated arginine-derived proline was rapidly incorporated into proteins rather than accumulating as a free metabolite, consistent with previous observations of a dissociation between intracellular proline levels and its availability for collagen synthesis^29^. Collagens make up a large fraction of the body mass (up to 12%) and they are proline-rich proteins^29–31^ that frequently increase in the interstitial during fibrotic response. Of note, this mouse model shows largely normal interstitial histology at 10 weeks of age with no fibrosis, despite marked proteinuria^15^. Therefore, we checked whether and to what extent collagens were labeled. With isotope-labeled dietary feeding, collagen labeling increased in the proteinuric kidneys compared with the controls: up to 10% of collagen was isotope-labeled with arginine and proline in control mice, compared with 18% in proteinuric mice (Fig. 4A, **Suppl. Table 17**), suggesting that proline is increasingly used for building collagens. In both the kidney cortex and medulla, the global labeling of collagens was markedly increased for both Arg7/Pro6 and Arg10/Pro6 labeling pairs (Fig. 4B, **Suppl. Table 18**) but also for each individual amino acid (Fig. 4C, Suppl. Fig. 3A, **Suppl. Table 18–19**). Of the 20 detected collagens, 11 showed significantly changed isotope incorporation, while the remaining collagens did not exhibit changes in labeling (Fig. 4D, Suppl. Fig. 3B, **Suppl. Table 20**). The collagens with increased incorporation included classical fibrillar and fibril-associated collagens (Col1a1, Col1a2, Col5a1, Col12a1, Col14a1), basement membrane-associated collagens (Col4a1, Col15a1, Col18a1), and pericellular collagens (Col6a1, Col6a2, Col6a3) (Fig. 4D, Suppl. Fig. 3B). Thus, in proteinuric conditions, dietary-derived arginine and proline are increasingly used to make collagens in the kidney.

### Proteinuria impairs ammonium disposal and remodels interorgan nitrogen handling

**Figure 5.**
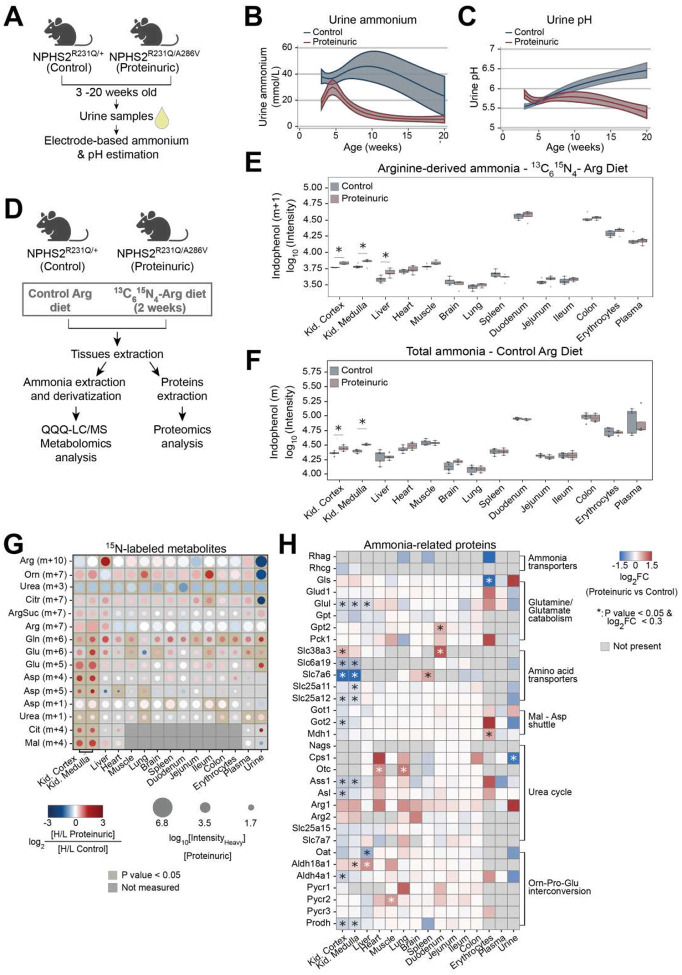
Altered interorgan arginine utilization is linked to reduced ammonium detoxification. **A.** Experimental workflow for ammonium and pH electrode-based measurement in control and proteinuric mice. **B-C.** Urine ammonium levels (**B**) and pH (**C**) in control (56 samples from 23 animals) and proteinuric mice (33 urine samples from 16 animals). **D.** Experimental workflow for ammonia estimation by HPLC LC/MS and proteomics analysis in control and proteinuric mice. **E-F.** System-wide differential abundance of labeled (**E**) and total (**F**) indophenol between control and proteinuric mice receiving the ^13^C_6_^15^N_4_-arginine diet and control arginine diet, respectively. Statistical significance was assessed with Student’s t-test (P < 0.05). **G.** System-wide differential abundance of ^15^N-labeled metabolites between control and proteinuric mice receiving the ^13^C_6_^15^N_4_-arginine diet. Statistical significance using a two-sided t-test (P < 0.05). **H.** System-wide differential abundance of arginine-related proteins between control and proteinuric mice receiving the control arginine diet. Asterisks indicate proteins with P < 0.05 and |log_2_ fold change| > 0.3.

The dietary isotope-labeling data suggested metabolic rewiring during proteinuric kidney disease, characterized by reduced commitment of dietary arginine to hepatic ureagenesis, increased renal use of arginine-derived carbon and nitrogen, a liver-derived albumin sink, and a kidney-derived proline/collagen sink. Because these pathways are tightly linked to nitrogen disposal, we next asked whether altered arginine fate in disease was accompanied by disrupted ammonium handling (Fig. 5A). We use “ammonium” throughout to refer to the pH-dependent NH_3_/NH_4_^+^ pool measured in urine and tissues. Urinary ammonium levels were reduced in proteinuric mice, indicating impaired net ammonium excretion in the setting of progressive disease (Fig. 5B, C, **Suppl. Table 21**). To determine whether tissue ammonia production was altered, ammonium was quantified by indophenol derivatization^14^ in tissue homogenates from mice fed an isotope-labeled arginine diet (Fig. 5D). Arginine-derived ammonium (indophenol m+1) was significantly increased in kidney cortex, kidney medulla, and liver, suggesting imbalanced urea cycle activity or altered ammonia handling in these tissues. (Fig. 5E, **Suppl. Table 22**). Total ammonia showed a similar trend in the kidney cortex and medulla (Fig. 5F, **Suppl. Table 22**). We next re-evaluated ^15^N-containing metabolites to assess inter-organ nitrogen redistribution. ^15^N-glutamine and ^15^N-glutamate were increased in skeletal muscle and kidney, and additional ^15^N signatures (including aspartate and urea isotopologues) were altered across tissues, with particularly strong labeling in the kidney (Fig. 5G). Detailed isotopologue assignments and quantification are provided in **Suppl. Table 23**. Finally, we examined tissue expression of ammonia-related enzymes and transporters (Fig. 5H). Glutamine synthetase (GLUL) was downregulated in the liver and kidney, the appropriate renal response to acid overload, while the basolateral glutamine transporter SLC38A3 was upregulated in the kidney cortex. In parallel, renal ASS1/ASL abundance was reduced, consistent with altered urea cycle enzyme activity in the kidney (Fig. 5H, **Suppl. Table 24**). Together, these findings indicate impaired ammonium disposal while promoting tissue accumulation of arginine-derived ammonium.

### Arginine-related aspartate modulates disease pathology

**Figure 6.**
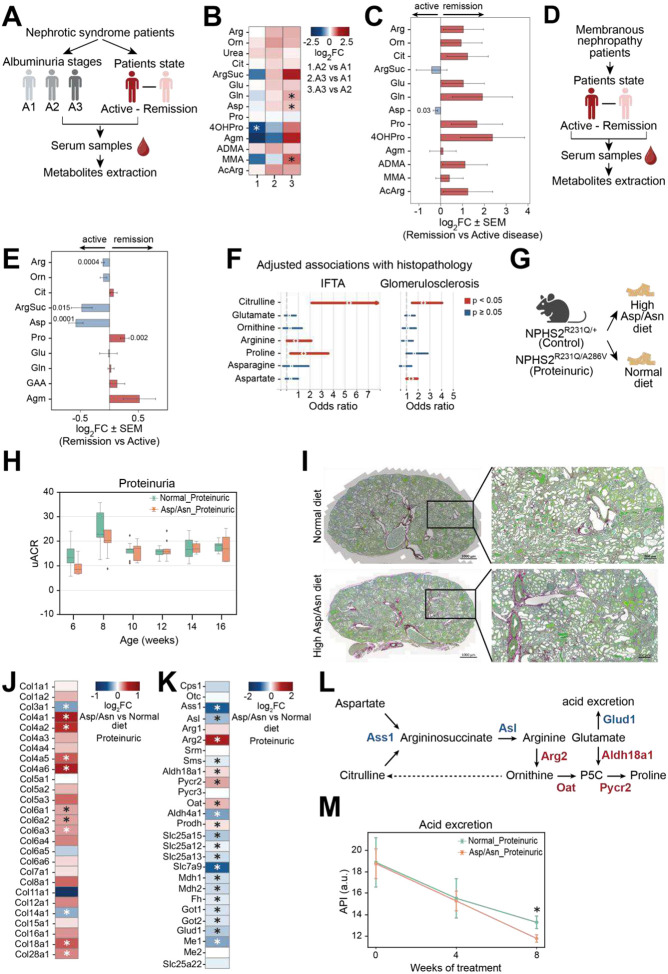
The arginine-related metabolite aspartate contributes to proteinuric kidney disease pathology. **A.** Experimental workflow for metabolite evaluation in the serum of patients with nephrotic syndrome. **B.** Abundance of arginine-related metabolites in serum of nephrotic syndrome patients of different albuminuria stages (KDIGO). Statistical significance was assessed using an unpaired two-sided Welch’s t-test (P value < 0.05). **C.** Paired changes in arginine-related metabolites between remission and active disease states in nephrotic syndrome patients with unchanged eGFR. Data shown as mean paired log_2_ fold changes (remission − active) ± SEM, one-sample t-test against zero. **D.** Experimental workflow for metabolites evaluation in serum of proteinuric membranous nephropathy patients in active disease and remission. **E.** Same as C for serum samples of membranous nephropathy patients. **F.** Association of metabolite signals for arginine metabolites with histology in the Boston Kidney Biopsy Cohort. The analysis reveals associations of citrulline, proline and arginine with interstitial fibrosis and tubular atrophy (IFTA), and association of aspartate and citrulline with glomerulosclerosis (corrected for age, sex, eGFR; race). **G.** Schematic of dietary intervention of control and proteinuric mice with a high aspartate/asparagine diet. **H.** Urinary albumin-to-creatine ratio (uACR) in proteinuric mice receiving a control or high aspartate/asparagine diet from 8 to 16 weeks of age (two-sided Mann–Whitney U test)). **I.** Histology images of mouse kidneys stained for fibrosis comparing normal and high aspartate/asparagine diet, scale bar 1000μm; inset 5x magnification, scale bar 200 μm. **J.** Differential abundance of collagens in control and proteinuric mice fed a control or high aspartate/asparagine diet. Statistical significance was assessed in Spectronaut DIA analysis. Asterisks show P value < 0.05. **K**. Same as J for arginine-related proteins. **L.** Schematic of regulated proteins and their function under high aspartate/asparagine diet. **M.** Ammonium-pH index indicating acid excretion between proteinuric mice receiving a control and a high aspartate/asparagine diet (asterisk: P value < 0.05, t-test).

To assess whether the arginine-linked metabolic change in mice were reflected in human proteinuric disease, we quantified arginine-related metabolites by targeted metabolomics in two independent patient serum datasets with preserved or stable GFR (Fig. 6A). In a cross-sectional selection of nephrotic syndrome patients, stratified by KDIGO albuminuria stage, patients with severely increased albuminuria (A3) exhibited higher serum levels of glutamine, aspartate, and monomethyl arginine than those with moderately increased albuminuria (A2) (Fig. 6B, **Suppl. Table 25**). In longitudinal paired serum samples from the same cohort, with preserved glomerular filtration rate (GFR) over time, aspartate levels were lower in remission than in the active state (Fig. 6C, **Suppl. Table 26**). This finding was replicated in an independent cohort of proteinuric membranous nephropathy patients (Fig. 6D), where aspartate levels were again higher in the active disease state (Fig. 6E, **Suppl. Table 27**). Furthermore, in proteinuric patients in the Boston Kidney Biopsy Cohort Study^32^, arginine, proline, aspartate and citrulline associated with histological markers of kidney fibrosis in biopsies (Fig. 6F) – both interstitial fibrosis and tubular atrophy (IFTA) and glomerulosclerosis - after adjustment for sex, age, race and GFR. These data support a dynamic association between circulating arginine metabolite levels and disease activity.

Because *in vivo* tracing and patient data implicated aspartate in proteinuric disease activity, we next tested whether increasing dietary aspartate availability altered disease progression in proteinuric mice. The formulated diet had double the content of aspartate, and additional asparagine that can be metabolized to aspartate (*please see methods for details*). Proteinuric NPHS2^R231Q/A286V^ and control NPHS2^R231Q/+^ mice were fed a diet enriched in aspartate and asparagine from 6 to 16 weeks of age (Fig. 6G). In the proteinuric mice, aspartate/asparagine supplementation did not affect proteinuria (Fig. 6H), but appeared to increase fibrosis (Fig. 6I, **Suppl. Table 28**), while also impairing survival (Suppl. Fig 4, **Suppl. Table 29**). Proteomic analysis of the kidney cortex was performed next. Analysis of collagen expression revealed, consistent with the fibrosis signal, a broad increase in various collagens in mice receiving the aspartate/asparagine-enriched diet (Fig. 6J, **Suppl. Table 31**), including the collagens with higher incorporation of arginine-derived amino acids (Fig. 4D). Consistently, the expression of various TCA cycle enzymes was decreased in the kidney of proteinuric mice receiving the aspartate-asparagine-enriched diet, while the urea-forming enzyme arginase 2 (ARG2) was increased (Fig. 6K, **Suppl. Table 32**). The proline synthesis enzymes, both derived from arginine and from glutamate, were increased, providing a correlate for increased collagen abundance (Fig. 6L). Across the proteome, not only collagens, but also proteins with high proline content were preferentially increased by the aspartate/asparagine-enriched diet compared with proteins enriched for other amino acids (Suppl. Fig. 5A–C, **Suppl. Tables 33–34**). Since Glud1, a glutamate enzyme essential for ammonium excretion was decreased, we also analyzed ammonium excretion under aspartate diet (Fig. 6L). The urine ammonium-pH index (API), an indicator of renal capacity for ammonium excretion^33^, was decreased in the late stages of the disease (Fig. 6M, **Suppl. Table 30**). Together, these data indicate that perturbation of the metabolic pathways identified by arginine isotope tracing may promote collagen accumulation, proline-rich protein remodeling, and impaired ammonium-linked acid handling in proteinuric kidneys.

## Discussion

Clinically, proteinuria is viewed as the consequence of glomerular barrier failure and considered a trigger and driver of kidney tubule injury through multiple direct or indirect mechanisms^34,35^. Protein loss has also been connected to altered liver protein synthesis in human and rodent studies^11,12,36,37^. Our data extend this decades-old data by demonstrating that sustained urinary protein loss causes a kidney-tubule-centered inter-organ metabolic response, instead of a broad compensatory increase in protein synthesis. Within this concept, analysis of the metabolic fate of arginine in both proteins and metabolites revealed a rerouting of arginine carbon and nitrogen away from canonical hepatic ureagenesis and toward renal proline- and aspartate-linked pathways.

Using dietary isotope labeling, we resolve the physiological fate of dietary amino acid arginine after two weeks of sustained labeling across liver, kidney, gut, plasma, and other tissues. This previously undescribed approach captures a time-integrated metabolic record of intestinal digestion, systemic precursor recycling, and protein incorporation with a depth and signal-to-noise ratio that acute tracer-infusion studies cannot achieve, as those typically produce only low-level labeling. The resulting signal is strong enough to quantify not only protein synthesis, but also the metabolic fate of protein-incorporated amino acids. In chronic proteinuric disease, Arg7 and Pro6 signatures revealed that a substantial fraction of protein-incorporated amino acids arises from metabolically reprocessed arginine pools rather than directly from the dietary precursor. Albumin, by contrast, is labeled predominantly from dietary arginine, identifying it as a sink for non-recycled arginine. This finding challenges the classical view that nephrotic adaptation is driven mainly by a generalized increase in hepatic protein synthesis to compensate for albumin loss. Instead, proteome-wide labeling remains largely buffered across organs, even though global incorporation declines in kidney cortex, brain, and intestine. Together, these data suggest that albumin loss in proteinuria is a major metabolic liability, diverting dietary arginine away from broader nitrogen-handling pathways and imposing a substantial penalty on nitrogen detoxification.

Mechanistically, the resulting metabolite isotope patterns point to altered whole-body arginine handling. Arginine occupies a central position at the intersection of the urea cycle, nitric oxide biology, creatine synthesis, and the ornithine–glutamate–proline axis, and the kidney is a major site of arginine generation from gut-derived citrulline through the intestinal–renal axis. The causative roles of altered and non-canonical extrahepatic arginine and urea cycle enzymes have recently been demonstrated in kidney and heart disease^38–41^. In proteinuria, dietary arginine accumulates in the liver with reduced downstream labeling of urea cycle metabolites, whereas the kidney selectively enriches arginine-derived glutamate, proline, and aspartate labeling. Although these readouts do not provide absolute fluxes, they are more consistent with reduced isotopic commitment of dietary arginine to hepatic ureagenesis and increased renal allocation to alternative arginine fates, indicating kidney–liver nitrogen miscoordination.

Our *ex vivo* nephron tracing and single-tubule proteomics^28^ localize this rewiring to the proximal nephron. The proximal tubule is the principal site of filtered-protein reclamation, a metabolically specialized segment, and a primary site of injury in proteinuric disease. In this compartment, tubular albumin is linked to enzymes involved in proline synthesis and nitrogen metabolism, including ALDH18A1, PRODH, and CPS1, suggesting that glomerular protein leak is translated into a defined tubular metabolic response, favoring proline synthesis – as shown by single-tubule proteomics and isotope tracing of microdissected oxygenated nephron segments. Thus, a major implication of this remodeling is that the proteinuric kidney becomes an early metabolic sink for arginine-derived proline. Collagen, a major extracellular matrix protein, is rich in proline and imposes a substantial biosynthetic demand as collagen formation is metabolically regulated by the environment^29^. In our model, arginine-derived proline is incorporated more rapidly into collagens in proteinuric mice. Interestingly, the accelerated synthesis occurs before the onset of prominent structural fibrosis, demonstrating that pre-fibrotic matrix anabolism is an early metabolic consequence of proteinuria. This provides a plausible direct biochemical link between tubular protein handling, amino acid rerouting, and progressive interstitial remodeling.

The cost of this altered nitrogen disposal and proline synthesis is reflected in ammonium retention. Ammonium is a toxic molecule linked to ATP depletion and oxidative stress^42,43^. The ammonium, detected through a direct derivatization method^14^, is isotope-labeled, meaning it derives directly from arginine, and is therefore linked to the altered glutamate generation. Impaired acid excretion increases glutamine uptake and glutamate recycling, which, in combination with the impaired capacity for ammonium excretion, causes ammonium to build up in the cells. This reroutes metabolism toward collagen formation via proline generation. Acid retention is a known consequence of end-stage kidney disease, in which the glomerular filtration rate is severely diminished. In this concept, we support the view that metabolic diseases of the tubule, independent of the GFR, can lead to acid retention in proteinuric kidney disease.

Patient data, with a GFR-independent increase in aspartate in the active proteinuria state, and association of studied metabolites align with several mouse findings and demonstrate excess aspartate in proteinuria (Fig. 6). These findings motivate deeper evaluation of amino acid–centered propagation of disease activity. Notably, aspartate is both the end and the starting point of a cycle that drives fibrosis, prompting us to feed excess amounts of the aspartate and also its precursor asparagine (for food formulation and nitrogen balance reasons). Together, kidney aspartate labeling, dynamic serum associations in two patient cohorts, and potential worsening of disease trajectories upon aspartate/asparagine supplementation suggest that aspartate can flood this system, forcing it into making proline and proline-derived proteins by increasing the already proline synthesis pathways in proteinuria (Fig. 6L).

Several limitations should be acknowledged. First, dietary isotopologue patterns do not directly yield absolute fluxes, and future studies integrating dietary tracing with formal flux analysis will be needed. Second, although the podocin-mutant model captures chronic proteinuria with important physiological advantages, metabolic adaptation may differ in inflammatory or diabetic glomerular disease. Third, although mice of both sexes were studied throughout, the studies were not designed to resolve sex differences, a known outcome mediator in kidney disease^44^. Nevertheless, the convergence of multi-organ tracing, nephron-segment metabolism, single-tubule proteomics, ammonia phenotyping, and human metabolomics supports a coherent model in which proteinuria acts as a chronic inter-organ nitrogen redistribution syndrome.

In summary, we propose that proteinuric kidney disease is a systemic metabolic state in which glomerular barrier failure redirects arginine metabolism, and potentially broader amino acid metabolism. This rerouting converts the proximal nephron into a pre-fibrotic proline/collagen sink, and impairs coordinated nitrogen and acid disposal across organs. This unified metabolic concept, revealed by a novel approach, helps explain how sustained proteinuria can drive tubulointerstitial remodeling and systemic complications out of proportion to the primary glomerular lesion, and suggests that targeting dietary amino-acid routing or kidney–liver nitrogen coordination may open therapeutic or preventative opportunities beyond conventional anti-proteinuria strategies.

## Detailed description of materials and methods

### Animal Studies

#### Ethics, housing, and generation of mouse lines.

All animal studies and procedures were performed according to the guidelines from Directive 2010/63/EU of the European Parliament on the protection of animals used for scientific purposes. The Animal Experiments Inspectorate of the Danish Ministry of Environment and Food approved the study #2018-15-0201-01601, #2023-15-0201-01585, #2021-15-0201-010743). Animal experiments were reported under the ARRIVE (Animal Research: Reporting *in vivo* Experiments) guidelines. All breeding pairs were housed in specific pathogen-free cages and received a fortified diet specifically designed for breeding mice (Altromin, Germany, 1318). The experimental animals were housed in individually ventilated cages. They received a fortified diet (Altromin, Germany, 1328) specifically designed for the maintenance of mice, unless a specific treatment was applied. All animals were housed under a 12:12-hour light–dark cycle, with a room temperature of 21–23°C and had free access to water through an automatic supply.

The experimental mouse lines *C57Bl/6N*-*NPHS2*A286VBsch/6NRj and *C57Bl/6N*-*NPHS2*R231QBsch/6NRj were generated with *in vitro* fertilization (IVF) in the Laboratory Animal Facility of the Department of Biomedicine of Aarhus University, following previously established research^45–48^. The cryopreserved sperm samples of both lines were obtained from the CECAD Research Center, University of Cologne, Germany^15^.

#### Breeding^15,45–48^

Male and female mice, 8 weeks old, were paired for natural mating. For the *C57Bl/6N*-*NPHS2*A286VBsch/6NRj line, progeny included control (“NPHS2^+/+^”), heterozygous (“NPHS2^A286V/+^”), and homozygous (“NPHS2^A286V/A286V^”) animals. For the *C57Bl/6N*-*NPHS2*R231QBsch/6NRj line, progeny included control (“NPHS2^+/+^”), heterozygous (“NPHS2^R231Q/+^”), and homozygous (“NPHS2^R231Q/R31Q^”) animals. Compound heterozygous NPHS2^R231Q/A286V^ (line *C57Bl/6N*-*NPHS2*<R231QBsch>/6NRj x *C57Bl/6N-NPHS2*<A286VBsch>/6NRj) were generated by crossing homozygous *C57Bl/6N*-*NPHS2*R231QBsch/6NRj mice with heterozygous *C57Bl/6N*-*NPHS2*A286VBsch/6NRj mice. For genotyping, DNA was extracted from tail or ear biopsies using the HotSHOT protocol^49^, and genotyping was performed using specific primers as described^15^.

### Dietary isotope labeling interventions

#### ^13^C_6_^15^N_4_-Arginine diet in wild-type mice.

Twenty-two 8-week-old *C57BL/6JRj* mice (female, n=11; male, n=11; strain name *C57BL/6JRj*, Janvier-Labs) were acclimatized for 1 week at the Laboratory Animal Facility, Department of Biomedicine, Aarhus University. After acclimatization, mice received either a custom ^13^C_6_^15^N_4_-arginine isotope-labeled diet (Arg(10)-SILAC-Mouse Diet; Silantes, Germany, 950005100, also referred to as the “^13^C_6_^15^N_4_-Arg diet”) for 1 week (n=6), 2 weeks (n=6), 8 weeks (n=4); or a control (unlabeled) mouse diet (Arg(0)-SILAC-Mouse Diet; Silantes, Germany, 950005100, also referred to as the “Control Arg diet”) for 8 weeks (n=6). Each group included equal numbers of male and female mice. Body weight was recorded weekly, and spot urine samples were collected three consecutive days prior to endpoint. Sample collection was performed as described below.

#### ^13^C_6_^15^N_4_-Arginine diet in NPHS2^R231Q/A286V^ mice.

Five NPHS2^R231Q/A286V^ (female, n=3; male, n=2) and six NPHS2^R231Q/+^ (female, n=3; male, n=3) mice, 8–weeks old, were fed a ^13^C_6_^15^N_4_-arginine diet (^13^C_6_^15^N_4_-Arg diet) for 2 weeks. Four NPHS2^R231Q/A286V^ (female, n=1; male, n=3) and 6 NPHS2^R231Q/+^ (female, n=3; male, n=3) 8–weeks old were fed a control arginine diet (Control Arg diet) for 2 weeks and served as controls. Body weight was recorded, and spot urine samples were collected weekly. Sample collection was performed as described below.

### High aspartate/asparagine diet supplementation of NPHS2^R231Q/A286V^ mice

Ten NPHS2^R231Q/A286V^ (female, n=5; male, n=5) and nine NPHS2^R231Q/+^ (female, n=5; male, n=4) were fed a custom Teklad 2020 diet enriched in aspartate (2.2%) and asparagine (1.1%) (“Asp/Asn diet”, Envigo, USA, TD.240403) compared with the baseline Teklad 2020 diet (“Normal diet”, Envigo, USA, TD.110180), for 11 weeks starting at 6–8 weeks of age. The asparagine is converted to aspartate through asparaginase activity (of the highly kidney expressed ASRLG1 enzyme). The diets were formulated by a mouse dietician according to practical and scientific considerations with some precedent for feeding high aspartate^50^, and were isocaloric. An equal number of NPHS2^R231Q/A286V^ and NPHS2^R231Q/+^ were fed a control Teklad 2020 mouse diet (Envigo, USA, TD.110180) for 11 weeks and served as controls. Body weight was recorded, and spontaneous urine samples were collected weekly.

### Sample collection and physiological measurements

#### Tissue, plasma, and urine collection.

At experimental endpoints, mice were anesthetized, blood was collected, and tissues were harvested as described below. In the ^13^C_6_^15^N_4_-arginine supplementation study in wild-type mice, mice were anesthetized by intraperitoneal injection of pentobarbital in sodium chloride 40 mg/mL. Blood was collected by cardiac puncture and centrifuged at 2000 × g at 4°C to separate plasma and erythrocytes. Mice were perfused with 5–10 mL ice-cold PBS (VWR Chemicals, USA, J373–4L). Tissues, including kidneys, liver, heart, muscle, brain, lung, spleen, duodenum, jejunum, ileum, and colon, were collected. Half of the kidney was fixed in OCT medium (Avantor, USA, 361603E), and the remaining parts of the kidney were dissected into the kidney cortex and medulla and stored separately. All samples were snap-frozen in liquid nitrogen. Spot urine was collected for two consecutive days before the endpoint and prior to anesthesia.

In the ^13^C_6_^15^N_4_-arginine supplementation study in NPHS2^R231Q/A286V^ mice, anesthesia was also performed by intraperitoneal injection of pentobarbital (40 mg/mL; Virbac, the Netherlands), with the dose adjusted according to body weight. Blood collection, perfusion, and tissue processing were performed as described for wild-type mice. Spot urine samples were collected weekly and prior to anesthesia.

In the high aspartate/asparagine diet study, mice were anesthetized with isoflurane. Blood was collected by puncture of the inferior vena cava and centrifuged at 1000 × g for 10 minutes at 4°C (Eppendorf 5430 R; Eppendorf, Germany) to separate plasma from erythrocytes. Mice were perfused with 5–10 mL ice-cold PBS (VWR Chemicals, USA, J373–4L). Kidney sections were fixed in 4% formaldehyde (Q Path, VWR, Poland, 11699455) and processed for paraffin embedding, while remaining tissues were snap-frozen in liquid nitrogen and stored at −80°C. Spot urine samples were collected every 2 weeks and prior to anesthesia. Urine samples were analyzed for creatinine and albumin levels as described below.

### Electrode-based urinary ammonium and pH measurements

Urinary ammonium and pH were measured longitudinally in control mice (56 urine samples from 23 animals) and proteinuric NPHS2^R231Q/A286V^ mice (33 urine samples from 16 animals), aged between 3 and 20 weeks. Control mice included NPHS2^R231Q/+^ and NPHS2^A286V/+^ animals, which were pooled for analysis. Urinary pH was measured using a micro pH-electrode (pH-500; Unisense, Aarhus, Denmark) and a reference electrode. Ammonium concentrations were measured using an ion-selective electrode (Orion^™^High-Performance Ammonia Ion-Selective Electrode, Thermo Scientific, Cat. No. 9512HPBNWP). Samples were alkalized with an ionic-strength adjusting solution (Thermo Scientific, Cat. No. 951 211), which converts ammonium (NH_4_^+^) to ammonia (NH_3_). Ammonia then diffuses through an ammonia-selective gas-permeable membrane, inducing a pH change that is detected by the electrode. The resulting voltage signal was quantified using a calibration curve. Changes in urinary NH_4_^+^ and pH were analyzed using mixed-effects models for repeated measures in StataBE 17.0 for Mac. Age, genotype, and the age-by-genotype interaction were included as fixed effects, and sex was included as a covariate. To account for within-mouse correlation, mouse ID was included as a random intercept, and age was modeled as a random slope. To account for potential non-linear relationships between age and the outcomes, age was parameterized using a restricted cubic spline with four knots.

### Determination of albumin-to-creatinine ratio and proteinuria measurements

The urine albumin levels were measured using the Albumin Blue Fluorescent Assay Kit (Active Motif, USA, 15002) according to the manufacturer’s instructions. Fluorescence was measured at an excitation wavelength of 560 nm and an emission wavelength of 620 nm using a SpectraMax iD3–3024 microplate reader (Molecular Devices, USA). Urinary creatinine levels were estimated using the Creatinine (Urinary) Colorimetric Assay Kit (Cayman Chemical, USA, 500701) according to the manufacturer’s instructions. Absorbance was measured at 495 nm using a BioTek PowerWave 340 microplate reader (BioTek, USA).

### Renal segments isolation

#### Glomeruli isolation from NPHS2^R231Q/A286V^ mice.

Four NPHS2^R231Q/A286V^ (female, n=2; male, n=2) and four NPHS2^R231Q/+^ (female, n=2; male, n=2), 8 weeks old, were euthanized by cervical dislocation, and the kidneys were removed along with the aorta. Kidneys were perfused through the renal arteries with 1 mL Dynabeads solution (Invitrogen, Norway, 14013), prepared by diluting 300 μL Dynabeads in 10 mL Hanks’ Balanced Salt Solution (“HBSS”; Gibco/Life Technologies, United Kingdom, 14065049). Kidneys were minced into approximately 1 mm^3^ pieces and digested with collagenase (1 mg/mL Collagenase, Type II; Gibco/Life Technologies, USA, 17101015) at 37°C for 15 minutes with gentle agitation. The digested tissue was passed through a 100 μm cell strainer (VWR, USA, 732–2759) using a flattened pestle into a 50 mL tube (Sarstedt, Germany, 62.547.254) and was washed with 5 mL HBSS. The suspension was subsequently filtered through a second 100 μm cell strainer and washed with up to 20 mL of HBSS. The filtrate was centrifuged at 1500 rpm for 5 minutes at 4°C (Centrifuge 5910R, Eppendorf, Germany). The pellet containing the glomeruli was resuspended in 1 mL HBSS and transferred to a 1.5 mL tube (Eppendorf, Germany, 0030120086). Glomeruli containing dynabeads were isolated using a magnetic particle concentrator and washed three times to remove remaining renal tubule fragments and tissue debris. During the procedure, kidney samples were maintained at 4°C except during Dynabead perfusion and collagenase digestion, which were performed at 37°C.

#### Enzymatic preparation of renal tubule suspensions from NPHS2^R231Q/A286V^ mice.

Four NPHS2^R231Q/A286V^ (female, n=2; male, n=2) and four NPHS2^R231Q/+^ (female, n=2; male, n=2), 8 weeks old, were euthanized by cervical dislocation, and the kidneys were quickly removed and placed in 98B buffer (NaCl (VWR Chemicals, Belgium, 27800.291), 0.4 mM K_2_HPO_4_ (VWR Chemicals, Belgium, 26932.290), 1.6 mM KH_2_PO_4_ (VWR Chemicals, Germany, 26923.298), 1 mM MgSO_4_ · 7H_2_O (VWR Chemicals, Belgium, 25167.298), 10 mM Na-acetate (VWR Chemicals, Belgium, 27653.235), 1 mM alpha-ketoglutarate (Acros Organics, United Kingdom, 271250500), 1.3 mM Ca-gluconate (Acros Organics, Italy, 211062500), 5 mM Glucose (VWR Chemical, Belgium, 101174Y), pH adjusted to 7.4 at 37°C). Renal cortical tissue was dissected, sliced and incubated in a preheated at 37°C incubation solution (1.3 mg DNAse I (Roche Diagnostics, Germany, 11284932001) and 19 mg Glycine (VWR Chemicals, Belgium, 101194M) dissolved in 50 mL 98B buffer and supplemented with 2 mg/mL collagenase Type II (Gibco (Life Technologies), USA, 17101–015). Digestion was performed in a ThermoMixer C (Eppendorf, Germany) at 37°C, with shaking at 850 rpm for 10 minutes. The resulting tubule suspension was transferred to a 2 mL tube (Sarstedt, Germany, 72.691) containing 1 mL sorting solution (25 mg albumin (Thermo Fisher Scientific, USA, BP9702–100) in 50 mL incubation) and placed on ice. Remaining tissue fragments were subjected to additional digestion by adding 1 mL prewarmed digestion solution and incubating at 37°C for 5 minutes. Newly released tubules were collected into separate tubes containing sorting solution on ice. This digestion step was repeated four times. To wash and remove erythrocytes and debris, tubule suspensions were allowed to sediment for 5 minutes, after which the supernatant was discarded and replaced with fresh sorting solution several times. Tubule suspensions from each mouse were pooled and aliquoted into equal volumes (10 μL per well) into V-bottom 96-well plates (Greiner, Germany, 651201).

#### Isolation of renal segments.

Four C57Bl6/J mice, 9 weeks old (female, n=2; male, n=2), were euthanized by decapitation. Enzymatic tubule preparation was performed as described above. Nephron segments, including glomeruli (n=30), proximal convoluted tubules (n=10), proximal straight tubules (n=10), thin ascending limbs of the loop of Henle (n=10), distal convoluted tubules (n=10) and collecting ducts (n=10) were manually isolated under a stereomicroscope (Leica M165C, Leica Microsystems, Germany) at 4°C based on their morphological characteristics^51^. Isolated nephron segments were incubated with either 0.155 mM ^12^C_6_-arginine (MilliporeSigma, USA, 101543) or 0.155 mM ^13^C_6_ arginine (Cambridge Isotope Laboratories, Inc., USA) for 30 minutes at 37°C with gentle agitation (350 rpm). The experiment was performed in duplicate. After incubation, samples were centrifuged at 14000 rpm for 10 minutes at 4°C, and the supernatant was discarded. The segment pellets were washed with 1x PBS (VWR Chemicals, USA, J373–4L), then centrifuged at 14000 rpm for 15 minutes at 4°C. The supernatant was discarded, and the washing step was repeated twice. For each incubation condition, two replicate series of segments were prepared per mouse. Pellets were stored at −80°C until metabolite extraction. Metabolites were extracted as described below.

#### Single proximal tubule isolation.

Three NPHS2^+/+^ animals (female, n=2) and two NPHS2^A286V/A286V^ (female, n=2), 11 weeks old, were euthanized by cervical dislocation. Kidneys were digested for enzymatic tubule preparation as described above. Single proximal tubules were manually isolated under a microscope at 4°C and transferred into wells of a 96-well plate.

### *Ex vivo* isotope labeling experiments

#### Incubation with isotope-labeled ^13^C_6_-arginine.

Isolated glomeruli and renal tubule suspensions were aliquoted (10 μL per well) into V-bottom 96-well plates (Greiner, Germany, 651201). Tubules were sufficiently oxygenated^22^. Samples were incubated in duplicate with either 0.155 mM ^12^C_6_-arginine (MilliporeSigma, USA, 101543), 0.155 mM ^13^C_6_-arginine (Cambridge Isotope Laboratories Inc., USA), or HBSS as a control for 15, 30, or 60 minutes. For manually isolated nephron segments, including glomeruli, proximal convoluted tubules, proximal straight tubules, thin ascending limbs of the loop of Henle, distal convoluted tubules, and collecting ducts, segments were incubated with either 0.155 mM ^12^C_6_-arginine or 0.155 mM ^13^C_6_-arginine for 30 minutes at 37°C with gentle agitation (350 rpm). The experiment was performed in duplicate. The purity of independent preparations was validated by proteomics analysis.

For glomeruli and tubules suspensions, reactions were terminated by adding 100 μL of ice-cold PBS (VWR Chemicals, USA, J373–4L), followed by centrifugation at 1000 × g for 1 minute at 4 °C (Centrifuge 5910R; Eppendorf, Germany). Supernatants were discarded, and samples were washed three times with ice-cold PBS. For nephron segments, samples were centrifuged at 14000 rpm for 10 minutes at 4°C, and the supernatant was discarded.

Pellets were washed with 1 × PBS, then centrifuged at 14000 rpm for 15 minutes at 4°C. The washing step was repeated twice. Pellets were stored at −80°C until metabolite extraction. Metabolites and proteins were extracted and further processed as described below.

### Human samples

#### Serum samples from nephrotic syndrome patients.

Serum samples were obtained from the National Unified Renal Translational Research Enterprise: Idiopathic Nephrotic Syndrome (NURTuRE-INS) study^52^. Samples were collected from 13 patients with nephrotic syndrome at two timepoints: baseline and six months later. For each sample, either the albumin-to-creatinine ratio (ACR) and/or the protein-to-creatinine ratio was available. Based on these measurements, samples were categorized according to the KDIGO-defined albuminuria stages (A1, A2, and A3). Finally, the combination of sampling time points and proteinuria status was used to categorize patient states as baseline-relapse or active-remission.

#### Serum samples from membranous nephropathy patients.

Serum samples from membranous nephropathy patients were collected at the National Institutes of Health, (NIH) Clinical Research Center, National Institute of Diabetes and Digestive and Kidney Diseases (NIDDK), Kidney Disease Section, under IRB-approved protocols. Research samples were collected from 42 patients with biopsy-proven membranous nephropathy at two timepoints: 1) “Active disease”: defined as urine ACR > 5000 mg/g or urine protein-to-creatinine ratio (PCR) > 5000 mg/g or 24-hour urinary protein > 5000 mg/day and serum albumin < 3 g/dl and eGFR > 60 ml/min, and 2) “Remission”: defined as urine ACR <1000 mg/g or urine PCR <1000 mg/g or 24-hour urinary albumin or protein < 1000 mg/day and eGFR > 60 ml/min.

#### Boston Kidney Biopsy Cohort.

The Boston Kidney Biopsy Cohort (BKBC) is a prospective, observational cohort study enrolling patients who underwent native kidney biopsy at three tertiary care hospitals in Boston, Massachusetts. Eligible participants were 18 years or older who had a clinically indicated kidney biopsy performed between September 2006 and October 2018. A detailed description of the study design has been published previously^32^. Blood samples from participants were provided on the day of kidney biopsy. Following collection, samples were stored at −80 °C until analysis. The Mass General Brigham Institutional Review Board (IRB) approved the BKBC study protocol. Kidney biopsy specimens were reviewed by two kidney pathologists using light microscopy. Each biopsy was assigned a semiquantitative score for interstitial fibrosis and tubular atrophy (IFTA) and global glomerulosclerosis, each scored as 0 (≤10%), 1 (11–25%), 2 (26–50%), or 3 (>50%)^32^. Metabolomic profiling was conducted on plasma samples obtained at the baseline visit of 444 BKBC cohort participants. For this analysis, we included 314 participants with ACR >= 300 mg/g and available metabolomic profiling. Metabolite quantification was performed by Metabolon, Inc. (Durham, NC) using ultrahigh-performance liquid chromatography-tandem mass spectrometry. Metabolite identification was based on retention index, chromatographic behavior, and mass-to-charge ratio, requiring agreement with authenticated standards within the Metabolon reference library.

#### Statistical analysis.

Plasma metabolites (aspartate, asparagine, proline, arginine, ornithine, glutamate, and citrulline) were log_2_-transformed prior to analysis. The association between each metabolite predictor and each binary histopathological outcome (score 0/1 vs. 2/3) was evaluated using unadjusted and multi-variable adjusted logistic regression models with adjustment for age, sex, race, and eGFR. Results are reported as odds ratios (OR) with 95% confidence intervals (CI) and two-sided p-values. A p-value of <0.05 was considered statistically significant. All analyses were performed in Stata 19 (StataCorp, College Station, TX).

### Metabolite extraction

#### Metabolites extraction from nephron segments.

Ice-cold metabolites extraction solution (100 μL) consisting of HPLC LC-MS grade acetonitrile (VWR, USA, 83640.320), methanol (VWR, USA, 85681.320), and water (VWR, France, 83645.320) in a 2:2:1 (v/v/v) ratio was added to the renal segments. Metabolite extraction was performed by three cycles of freezing in liquid nitrogen followed by thawing with sonication in an ultrasonic bath at 4°C for 15 minutes. Samples were then incubated at −20°C for 2 hours. Extracts were centrifuged for 20 minutes at 4°C at 16000 × g in Eppendorf tubes or at 4347 × g in 96-well plates. The supernatants containing metabolites were transferred to new V-bottom-shaped 96-well plates. Proteins were subsequently extracted from the remaining tissue pellets, as described below, and protein concentration was determined using the BCA assay (Company, Country, A55864). Metabolite extracts were dried overnight in a SpeedVac concentrator (Labconco Centrivap) at 8°C. Dried metabolites were resuspended in a 1:1 (v/v) mixture of HPLC LC-MS grade acetonitrile (VWR, USA, 83640.320) and water (VWR, France, 83645.320) with volumes normalized according to the protein content determined by the BCA assay. Samples were centrifuged at 4347 rcf for 1 hour at 4 °C (Centrifuge 5910 R, Eppendorf, Germany), and the supernatants were transferred to a new V-bottom 96-well plate. Extracts were stored at −80°C or immediately subjected to LC-MS analysis for targeted metabolomics.

#### Metabolites extraction from tissues.

Frozen organs were maintained on dry ice, and approximately 10 mg of tissue was weighed for metabolite extraction. Ice-cold extraction solution (800 μL) consisting of HPLC LC-MS grade acetonitrile (VWR, USA, 83640.320), methanol (VWR, USA, 85681.320), and water (VWR, France, 83645.320) in a 2:2:1 (v/v/v) ratio, was added together with two stainless steel beads (Next Advance, USA, SSB32). Samples were homogenized using a Bullet Blender Storm Pro (Next Advance, USA) at speed 8 until complete tissue disruption. Tissue homogenates were incubated at −20°C for 2 hours to facilitate metabolite extraction. Samples were then centrifuged (Centrifuge 5430 R; Eppendorf, Germany) at 16000 × g for 20 minutes at 4°C. The supernatants were transferred to 1.5 mL Eppendorf tubes (Eppendorf, Germany, 0030120086) on ice and dried overnight in a SpeedVac concentrator (Labconco Centrivap) at 8°C. Dried extracts were resuspended in 150 μL per 10 mg tissue of a 1:1 (v/v) mixture of HPLC LC-MS grade acetonitrile (VWR, USA, 83640.320) and water (VWR, France, 83645.320) and sonicated for 10 minutes at 4°C. Samples were centrifuged again at 16000 g for 20 minutes at 4°C, and the supernatants were transferred to mass spectrometry vials (VWR, Germany, 548–0029a) or a V-bottom 96-well plate (Greiner, Germany, 651201) and stored at −80°C until analysis. Quality control samples were prepared by pooling 5 μL from each sample.

#### Urine/plasma metabolites extraction.

Frozen plasma and urine samples were thawed on ice. Metabolites were extracted by adding ice-cold extraction buffer consisting of 80% HPLC LC-MS grade methanol (VWR, USA, 85681.320) and 20% HPLC LC-MS grade water (VWR, France, 83645.320) at a sample-to-buffer ratio of 1:4. Samples were incubated on dry ice for 2 hours to facilitate metabolite extraction. The extracts were then centrifuged at 16000 × g for 20 minutes at 4°C. The supernatants containing metabolites were transferred to new 1.5 mL Eppendorf tubes and stored at −80°C until further analysis.

### Ammonium derivatization

#### Ammonium extraction for LC-MS.

Frozen organs were maintained on dry ice, and approximately 10 mg of tissue was weighed. Extraction solution (400 μL per 10 mg tissue) consisting of HPLC LC-MS grade methanol (VWR, USA, 85681.320) and HPLC LC-MS grade water (VWR, France, 83645.320) in a 4:1 (v/v) ratio was added together with two stainless steel beads. Samples were homogenized using a bullet blender at 1–2-minute intervals at speed 8–10 until complete tissue disruption. Metabolite extraction was performed by incubating the homogenates on dry ice for 2 hours. Samples were then centrifuged at 16000 × g for 20 minutes at 4°C, and the supernatants were transferred to new 1.5 mL Eppendorf tubes. Ammonia extracts were stored at −80°C until further derivatization. For ammonia extraction from biofluids, the same extraction procedure was used, except that samples were vortexed for 10 seconds instead of homogenized with the bullet blender.

#### Ammonium derivatization.

Ammonia derivatization was performed as previously described^53^. Briefly, two derivatization solutions were prepared freshly on the day of the experiment. Solution 1 consisted of 250 mM Sodium hydroxide (VWR Chemicals, USA, 0583–500G) and 10 mM Dibasic sodium hydrogen phosphate (VWR Chemicals, Belgium, 28026.260) in HPLC LC-MS grade water (VWR, France, 83645.320), supplemented with 10% sodium hypochlorite (Thermo Scientific Chemicals, USA, 10401841). Solution 2 consisted of 99% 1M phenol (Sigma Aldrich, USA, 33517) in ethanol (VWR Chemicals, USA, 20821.296) and 1% of a 0.5% (w/v) sodium nitroprusside (Supelco, USA, 106541) prepared in HPLC LC-MS grade water (VWR, France, 83645.320).

Ammonia extracts were diluted 1:10 in extraction solution consisting of HPLC LC-MS grade methanol (VWR, USA, 85681.320) and HPLC LC-MS grade water (VWR, France, 83645.320) in a 4:1 (v/v) ratio. Subsequently, 100 μL of Solution 1 and 100 μL of Solution 2 were added to each diluted sample, while keeping the samples on ice. Samples were vortexed for 10 seconds and incubated in a ThermoMixer C (Eppendorf, Germany) at 37°C and 350 rpm for 30 minutes to allow ammonia derivatization. After derivatization, samples were transferred to mass spectrometry vials and analyzed directly by LC-MS. A negative control consisting of 20 μL extraction solution and a positive control consisting of 10% of a 100 mM indophenol solution in extraction buffer were included. Quality control samples for each tissue or biofluid type were prepared by pooling 3 μL from each sample.

### Targeted metabolomics LC-MS analysis.

Targeted metabolomic analysis was performed using a triple quadrupole (QQQ) mass spectrometer (Agilent Triple Quadrupole 6495C, San Diego, USA), coupled to an ultra-high pressure liquid chromatography system (UPLC) system (1290 Infinity, Agilent Technologies). Data acquisition was carried out using Agilent MassHunter Workstation Data Acquisition software (version 10.1).

#### LC–MS analysis of arginine-related metabolites.

Metabolites separation was performed using an Acquity UPLC BEH Amide column (1.7 um, 2.1 × 100 mm) (Waters, Ireland, 186002352). Collision energies and product ions (MS2 or quantifier and qualifier ion transitions) were optimized. Electrospray ionization (ESI) source parameters were set as follows: gas temperature 210°C, gas flow 20 L/min, Nebulizer 30 psi, sheath gas temperature 350°C, capillary voltage 1500 V, and nozzle voltage/charging 1000 V. For liquid chromatography, mobile phase consisted of 20 mM formic acid ammonium salt (VWR Chemicals, United Kingdom, 84884.180) and mobile phase B consisted of 99.9% HPLC LC-MS grade acetonitrile (VWR Chemicals, USA, 83640.320) with 0.1% formic acid (VWR Chemicals, United Kingdom, 84865.260). The gradient program (A/B) was as follows: 0 min, 5/95; 1.5 min, 5/95; 17 min, 55/45; 19 min, 55/45; 19.10 min, 5/95. The flow rate was 0.4 ml/min, and the injection volume was 2 μL. Dynamic multiple reaction monitoring (MRM) mode was used for metabolite detection. The list of targeted metabolites, together with their precursor/product ion transitions and collision energies, are provided in **Supplementary Table 35**.

#### LC–MS analysis of TCA cycle and Gluconeogenesis metabolites.

Metabolite separation was performed using an InfinityLab Poroshell 120 HILC-Z column (2.7 um, 2.1 × 150 mm) (Agilent, San Diego, CA). Collision energies and product ions (MS2 or quantifier and qualifier ion transitions) were optimized for each metabolite. Electrospray ionization (ESI) source parameters were set as follows: gas temperature 200°C, gas flow 15 L/min, Nebulizer 25 psi, sheath gas temperature 325°C, capillary voltage 3000 V, and nozzle voltage/charging 1500 V. For liquid chromatography, mobile phase A consisted of 20 mM ammonium acetate (VWR Chemicals, USA, 84885.180) in HPLC LC-MS grade water (VWR Chemicals, France, 83645.320) adjusted to pH 9.7, and mobile phase B consisted of 100% HPLC LC-MS grade acetonitrile (VWR Chemicals, USA, 83640.320). The gradient program (A/B) was as follows: 0 min, 10/90; 1.5 min, 10/90; 8 min, 50/50; 11 min, 50/50; 12 min, 55/45; 15 min, 55/45; 15.10 min, 10/90. The flow rate was 0.4 mL/min, and the injection volume was 2 μL. Dynamic multiple reaction monitoring (MRM) was used for metabolite detection. The list of targeted metabolites, together with their precursor/product ion transitions and collision energies, is provided in **Supplementary Table 35**.

#### LC–MS analysis of indophenol.

Metabolite separation was performed using an Acquity UPLC BEH C18 column (1.7 um, 2.1 × 100 mm) (Agilent, San Diego, CA). Collision energy and product ion (MS2 or quantifier and qualifier ion transitions) were optimized. Electrospray ionization (ESI) source parameters were set as follows: gas temperature 150°C, gas flow 11 L/min, Nebulizer 40 psi, sheath gas temperature 400°C, capillary voltage 3000 V, and nozzle voltage/charging 1500 V. For liquid chromatography, mobile phase A consisted of 97% HPLC LC-MS grade water (VWR Chemicals, France, 83645.320) and 3% HPLC LC-MS grade methanol (VWR Chemicals, USA, 85681.320) containing 15 mM acetic acid (Sigma-Aldrich, USA, 33209-M). Mobile phase B consisted of 100% HPLC LC-MS grade methanol (VWR Chemicals, USA, 85681.320) containing 15 mM acetic acid (Sigma-Aldrich, USA, 33209-M). The gradient program (A/B) was as follows: 0 min, 99/1; 1 min, 99/1; 6 min, 40/60; 7 min, 1/99; 10 min, 1/99; 10.10 min, 99/1. The flow rate was 0.45 mL/min, and the injection volume was 5 μL. Multiple reaction monitoring (MRM) was used for metabolite detection. The precursor/product ion transitions and the collision energy are provided in **Supplementary Table 35**.

### Proteomics sample preparation and LC-MS analysis

#### Proteomics sample preparation.

Protein extraction was performed either from intact tissue pieces or from tissue pellets remaining after metabolite extraction. Tissue pieces (20 mg) were homogenized in 300 μL extraction buffer at 4°C using a bullet blender (Bullet Blender Storm Pro; Next Advance, USA) for 3 minutes at speed 8, with 2 steel beads per sample (Next Advance, USA, SSB32). The extraction buffer contained 4% SDS (Sodium dodecyl sulfate) (Sigma Aldrich, Germany, 75746), 0.1 M HEPES (4-(2-Hydroxyethyl)piperazin-1-ylethanesulphonic acid) (VWR Chemicals, Belgium, 441485H), and 2.5 mM EDTA (Ethylenediamine tetra-acetic acid) (VWR Chemicals, Belgium, 280214S), supplemented with protease inhibitors (Complete; Roche Diagnostics, Germany, 05 056 489 001). Homogenized tissues or tissue pellets were incubated in extraction buffer (300 μL for tissue pellets and 60 μL for renal segment pellets) at 95°C for 10 minutes using a thermomixer (ThermoMixer C; Eppendorf, Germany). After centrifugation, the supernatants containing the lysed proteins were transferred to new tubes or 96-well plates. An aliquot of each lysate was diluted for protein concentration determination using a bicinchoninic acid assay (Thermo Scientific; USA, A55864). The remaining protein lysates were treated with 26 U benzonase (EMD Millipore, Denmark, 70664) and incubated at 37°C for 30 minutes. Samples were then sequentially treated with 10 mM DTT at 37°C for 30 minutes, followed by 50 mM IAA or CAA at room temperature in the dark for 30 minutes, and subsequently with 50 mM DTT at room temperature for 20 minutes. Proteins were bound to SP3 beads by adding 1 μL bead stock (1:1 mixture of hydrophilic beads (Cytiva, USA, 45152105050250) and hydrophobic beads (Cytiva, USA, 65152105050250)) per 20 μg protein, together with 100% HPLC quality ethanol (VWR Chemicals, USA, 20821.296) to final ethanol concentration of 80% and incubated at room temperature for 18 minutes without shaking. Beads were collected on a magnetic stand and washed twice with 400 μL 90% EtOH in LC-MS grade water (VWR Chemicals, France, 83645.320). After washing, the residual liquid was completely removed. The bead-bound proteins were re-suspended in 30 μL digestion buffer containing 50 mM HEPES pH 7.5 (VWR Chemicals, Belgium, 441485H), 5 mM CaCl_2_ (VWR Chemicals, Belgium, 22328.262), and MS-grade trypsin (Serva, Germany, 003728603) at a 1:100 protease-to-protein ratio and digested overnight at 37°C. After digestion, the peptide solution was acidified to pH < 3 by adding 25% formic acid (VWR Chemicals, United Kingdom, 84865.260) to a final concentration of 1–2%. Peptides were purified using reverse-phase C18 disks (Empore; Dr. Maisch), dried in a SpeedVac (Labconco Centrivap) and reconstituted in 0.1% formic acid in LC-MS grade water (VWR Chemicals, France, 83645.320). Peptide concentrations were determined using a Nanodrop spectrophotometer.

#### Nano-LC/MS proteomic analysis.

The purified peptides were analyzed using a Thermo UltiMate3000 rapid separation liquid chromatography (LC) system (Thermo Fisher Scientific) coupled to an Orbitrap Exploris 480 mass spectrometer equipped with a high-field asymmetric waveform ion mobility spectrometry (FAIMS) interface (Thermo Fisher Scientific). Peptides were separated using a two-column setup consisting of a trap column (Acclaim PepMap 100 C18, 3 μm particle size, 2 cm length, 75 μm inner diameter; Thermo Fisher Scientific) operated at a flow rate of 5 μL/min and an analytical column (Aurora 25 cm × 75 μm; IonOpticks) operated at a flow rate of 400 nL/min at 50°C.

#### Data-dependent acquisition (DDA).

Data-dependent acquisition (DDA) was used for the identification of peptide modifications. Peptides were separated using a 150-minute LC gradient with mobile phase A consisting of 0.1% formic acid in LC–MS–grade water and mobile phase B consisting of 0.1% formic acid in LC–MS–grade acetonitrile. The gradient started at 2% buffer B for 5 minutes, followed by a linear increase to 10% B over 10 minutes, and then to 50% B over 110 minutes. At 126 minutes, the gradient was increased to 90% B, followed by column equilibration from 136 to 150 minutes at 2% B. MS measurements were performed in positive ion mode using three FAIMS compensation voltages: −40 V, −55 V, and −70 V. Cycle times were 1.5 seconds for −40 V, and 0.75 seconds each for −55 V and −70 V. MS1 spectra were acquired in profile mode at a resolution of 120 000 over a scan range of 350–1350 m/z, with a normalized automatic gain control target of 300%, an RF lens setting of 40%, charge states from 2 to 6 included, a minimum precursor intensity of 2.5 × 10^4^, dynamic exclusion of 40 seconds, and automatic maximum injection time. MS2 spectra were acquired in centroid mode at a resolution of 15000 using higher-energy collisional dissociation with a normalized collision energy of 28%. The isolation window was set to 0.2 m/z, with a normalized automatic gain control target of 100%, a fixed first mass of 110 m/z, and automatic maximum injection time.

#### Data-independent acquisition (DIA).

Data-independent acquisition (DIA) was performed for high-sensitivity proteome measurements using a 120-minute LC gradient with mobile phase A consisting of 0.1% formic acid in LC-MS-grade water and mobile phase B consisting of 0.1% formic acid in LC-MS-grade acetonitrile. The gradient started at 2% B for 5 minutes, followed by a linear increase to 8% B over 5 minutes and then to 25% B over 80 minutes. This was followed by a step to 35% B over 10 minutes. At 101 minutes, the gradient was increased to 90% B, followed by column equilibration from 110 to 120 minutes at 2% B. All MS measurements were performed in positive ion mode using two FAIMS compensation voltages: −45 V and −65 V. MS1 spectra were acquired in profile mode at a resolution of 120000 over a scan range of 380–1500 m/z. MS2 spectra were acquired with a normalized automatic gain control target of 1000%, an RF lens setting of 40%, and a precursor mass range of 400–1000 m/z. DIA scans were recorded using non-overlapping isolation windows of 15 m/z with a normalized collision energy of 28%, in profile mode at a resolution of 20000^54,55^.

#### Spectronaut analysis of DIA data.

RAW MS files were analyzed using Spectronaut version 19.1 (Biognosys) with the directDIA+ (Deep) workflow. For Pulsar search, trypsin/P was specified as the digestion enzyme, with up to two missed cleavages allowed. The minimum peptide length was set to 7 amino acids, and the maximum peptide length to 52 amino acids. Labeling parameters were disabled for all analyses, except for searches investigating the incorporation of pairs of isotope-labeled amino acids. Carbamidomethylation of cysteine was specified as a fixed modification, while methionine oxidation and protein N-terminal acetylation were included as variable modifications. The false discovery rate for peptide-spectrum matches, peptides, and protein groups was controlled at 1%. For post-analysis settings, group-wise testing correction was applied. Liver, medulla, and cortex datasets from control (NPHS2^R231Q/+^) and proteinuric (NPHS2^R231Q/A286V^) were searched against a *UniProt Mus musculus* canonical database including both reviewed and unreviewed entries (downloaded June 28, 2023; 55,275 entries). Datasets from all other tissues of these mice were searched against a *UniProt Mus musculus* reference and model proteome database (downloaded January 22, 2025; 54,742 entries).

#### Spectronaut analysis for detection of isotope-labeled amino acid incorporation.

To investigate the incorporation of isotope-labelled amino acids Arg10, Arg7, and Pro6 into proteins in tissues from mice fed the ^13^C_6_^15^N_4_-Arg diet or the Control Arg diet, Spectronaut analysis was performed as described above, with modified labeling parameters in the Pulsar search. Channel 1 was left empty, Channel 2 included the labels Arg10 (mass shift 10.01 Da) and Pro6 (mass shift 6.01 Da), and Channel 3 included the labels Arg7 (mass shift 7.02 Da) and Pro6 (mass shift 6.01 Da). This analysis identified proteins in which both labeled amino acids of each pair were detected. Datasets from colon and erythrocytes were searched against a UniProt *Mus musculus* reference proteome database (downloaded January 22, 2025; 54742 entries), whereas datasets from all other tissues were searched against a UniProt *Mus musculus* proteome database (downloaded June 28, 2023; 55275 entries), both including reviewed and unreviewed entries.

#### FragPipe analysis of single tubule proteome data (DIA).

Mass spectrometry runs were queried with FragPipe v20.0 with the integrated DiaNN v1.8.1beta2^56^ using the default DIA_SpecLib_Quant workflow for library-free DIA analysis. Searches were performed with tryptic specificity allowing up to one missed cleavage against the canonical UniProt mouse reference proteome (release 2023–04; 17136 entries), supplemented with a corresponding reverse decoy database. Carbamidomethylation of cysteine residues (+57.02146 Da) was specified as a fixed modification, while oxidation of methionine (+15.9949 Da) and protein N-terminal acetylation (+42.0106 Da) were included as variable modifications. All other parameters were kept in default settings.

#### FragPipe open search for untargeted identification of PTMs on amino acids (DDA data).

To identify amino-acid mass shifts in peptides derived from mice fed a ^13^C_6_^15^N_4_-arginine diet for 2 weeks, an open search was performed using FragPipe v. 21.1 with MS Fragger 4.0 on data which was acquired with DDA. Searches were conducted using default parameters with a precursor mass tolerance window of −150 to +500 Da and trypsin/P specificity with a maximum of two missed cleavages. Carbamidomethylation of cysteine residues was set as a fixed modification, while methionine oxidation and protein N-terminal acetylation were included as variable modifications. Peptide length was restricted to 7 – 50 amino acids with a peptide mass range of 500 – 5000 Da. Database searches were performed against the canonical UniProt mouse reference proteome (release 2023-04; file: 2023-04-25-decoys-reviews-contam-UPS0000589), downloaded on April 25, 2023, containing 17136 protein entries.

### Statistical and bioinformatic analysis

#### Data visualization, statistics, and graphical representations.

Data visualization and statistical analysis were performed using Python (v3.9)^57^ with the following packages: seaborn (v.0.11.2)^58^, matplotlib (v3.7.0)^59^, pandas (v2.2.3)^60–62^, scipy (v1.10.1)^63,64^, numpy (v1.24.2)^65–67^, lifelines. scikit-learn Generative AI tools (ChatGPT), were used to assist with code generation and refinement. Graphical elements were created using icons from BioRender.com, and further processed in Adobe Illustrator^68^.

#### Differential expression analysis of proteins from tissues of NPHS2^R231Q/A286V^ mice.

Differential expression analysis was performed for each organ separately using the limma package (v.3.62.2)^69^. Normalized protein expression values were log_2_-transformed prior to analysis. Linear models were fitted for each protein using the lmFit function, followed by empirical Bayes moderation of the standard errors using eBayes. The sample size was n=6 for control and proteinuric mice per tissue, except for kidney cortex (n=5 and n=4, respectively) and urine (n=4 and n=3, respectively). Pancreas and white adipose tissue were excluded from the analysis due to the low number of detected proteins. Proteins were considered differentially expressed if they met the thresholds of an absolute log_2_ fold change > 0.3 and a limma-moderated P value < 0.05. For heatmap visualization, a subset of arginine-related proteins was selected from the LIMMA results. When multiple protein entries corresponded to the same gene, canonical UniProt identifiers were prioritized to resolve duplicates.

#### Differential abundance of collagens and arginine-related proteins in control and proteinuric mice fed either a control or a high aspartate/asparagine diet.

Differential protein abundance was calculated in Spectronaut (Biognosys) as part of the DIA proteomics analysis. Proteins supported by fewer than three unique peptides were excluded. For selected collagen proteins, abundance values were summarized across the four experimental groups as mean ± SEM based on protein quantity values.

#### Functional enrichment analysis.

Functional enrichment analysis of differentially expressed proteins from tissues of control NPHS2^R231Q/+^ and NPHS2^R231Q/A286V^ mice was performed using the STRINGdb R package (v.2.18.0). Differentially expressed proteins from each tissue were mapped to STRING identifiers using the built-in mapping functions. The sample sizes were n=6 control and n=6 proteinuric mice per tissue, except for the kidney cortex (control n=5, proteinuric n=4), and urine (control n=4, proteinuric n=3). Enrichment analysis was conducted using the get_enrichment() function, querying the STRING database (version 12) for pathway annotations (e.g., KEGG, Reactome, Wikipathways, and MPO) as well as GO categories, including Biological Process, Molecular Function and Cellular Component. Enrichment results were filtered using a false discovery rate (FDR) threshold of 0.05. The three most significantly enriched terms were selected and used for subsequent enrichment analysis across tissues. All analyses were performed in R (v4.4.3) using the RStudio environment (v2025.05.0)^70,71^.

#### Enrichment estimation and PSM-based quantification of labeled amino acids.

The enrichment percentage was calculated as the number of amino-acid-specific localized peptide–spectrum matches (PSMs) divided by the total number of localized PSMs for the respective mass shift. This calculation was performed with cortex and medulla samples from control, proteinuric, and wild-type mice fed either the control or arginine-labeled diet. Two cortex samples with uncertain genotypes were also included in this analysis. Entries annotated as “First isotopic peak” were excluded, and the three most enriched labeled amino acids were selected for visualization. Labeled amino acids were quantified based on the number of peptide–spectrum matches (PSMs). Two samples with uncertain genotypes were excluded from this quantification step, which did not affect the results.

#### Protein abundance estimation with and without incorporation of labeled amino acids.

Labeled amino acid incorporation across tissues was quantified using MS2 channel intensities obtained from Spectronaut DIA analysis. Mice received either a ^13^C_6_^15^N_4_-arginine diet or a control arginine diet. For mice receiving the ^13^C_6_^15^N_4_-arginine diet, the sample size was n=6 control mice and n=5 proteinuric mice per tissue, except for the kidney cortex (control n=5, proteinuric n=4). For mice receiving the control arginine diet, the sample size was n=6 control mice and n=4 proteinuric mice per tissue, except for kidney cortex (control n=5, proteinuric n=4) and urine (control n=4, proteinuric n=3). Pancreas and white adipose tissue were excluded from the analysis due to the low number of detected proteins. For each tissue and condition, intensities from the three MS2 channels corresponding to unlabeled peptides (Channel 1) and labeled amino acids (Arg10/Pro6, Channel 2; Arg7/Pro6, Channel 3) were summed at the protein level for each individual sample. Values were then averaged across mice within each condition to obtain mean channel intensities per tissue. For statistical analysis, summed MS2 channel intensities were compared between control and proteinuric mice within each of the control and arginine diet groups. Statistical significance was assessed using two-sided Welch’s t-tests. Relative incorporation of labeled amino acids was calculated by normalizing channel intensities to the total signal across the three channels for each condition, yielding percentage contributions of unlabeled and labeled peptides. Absolute summed MS2 channel intensities and the percentage contribution of each channel to the total signal were visualized across tissues. The same analysis workflow was additionally applied to albumin and to all detected collagen proteins across tissues. For these analyses, incorporation estimates were also recalculated using alternative channel assignments. For collagen proteins and albumin, labeling was additionally evaluated using channel assignments corresponding to Arg10 in Channel 2 and Pro6 in Channel 3, or as Arg7 in Channel 2 and Pro6 in Channel 3, as indicated in the text. In the manuscript, results for albumin and collagen proteins are shown for mice receiving the ^13^C_6_^15^N_4_-arginine diet.

#### Collagen channel-intensity analysis in kidney cortex and medulla.

To assess labeled amino acid incorporation into collagen proteins in the kidney cortex and medulla of mice receiving the ^13^C_6_^15^N_4_-arginine diet, MS2 channel intensities corresponding to Channel 2 (Arg10/Pro6), and Channel 3 (Arg7/Pro6) were obtained for all detected collagen proteins and summed across proteins for each samples. These values were then aggregated at the individual mouse level, such that each data point represented the summed collagen channel intensity from one mouse. Comparisons between control and proteinuric mice were performed using a two-sided Mann–Whitney U test. The same analysis was additionally performed using an alternative channel assignment in which Channel 2 corresponded to either Arg10 or Arg7 and Channel 3 to Pro6 labeling.

#### Collagen differential abundance analysis.

Differential abundance between control and proteinuric mice was assessed using Spectronaut searches, which provided log_2_ fold changes and associated P values for each protein. Collagen proteins enriched in Arg10 and Pro6 were analyzed, and the same analysis was performed for those enriched in Arg7 and Pro6. Proteins with P < 0.05 were annotated with an asterisk.

#### Correlation of labeling ratios with protein abundance changes.

Spearman correlation coefficients were calculated to assess the relationship between changes in protein abundance and isotope labeling enrichment. Protein abundance changes were expressed as log_2_ fold changes between proteinuric and control mice under the control arginine diet. Labeling enrichment was quantified using labeled-to-unlabeled peptide ratios (Arg10/Pro6 or Arg7/Pro6 relative to unlabeled peptides). The sample size was n=6 control mice and n=6 proteinuric mice per tissue, except for the kidney cortex (control n=5, proteinuric n=4) and urine (control n=4, proteinuric n=3). Pancreas and white adipose tissue were excluded from the analysis due to the low number of detected proteins. For tissue-level comparisons, Spearman correlation coefficients between labeling enrichment and protein log_2_FC were calculated for each organ. Correlation coefficients obtained from Arg10/Pro6 labeling and Arg7/Pro6 labeling were plotted against each other to compare labeling behavior across tissues. In addition, the analysis was repeated for a subset of plasma proteins with molecular weight <70 kDa, based on UniProt annotations, to assess whether protein size influenced labeling–abundance relationships. For this subset, proteins were required to be quantified across all relevant comparisons, and correlations between abundance changes and labeling differences were evaluated.

#### Differential abundance analysis of unlabeled and isotope-labeled metabolites.

For isotope-labeled metabolites, differential abundance was calculated as the difference between the log_2_-transformed mean heavy-to-unlabeled ratio in proteinuric and control mice fed the arginine-labeled diet. Statistical significance was assessed using a two-sided t-test (P < 0.05). For indophenol detected in mice receiving the arginine-labeled diet, differential abundance was calculated using log_10_-transformed peak areas of the labeled indophenol signal. Statistical significance was assessed using Student’s t-test (P < 0.05). For the analysis of unlabeled metabolites, the sample size was n=6 control mice and n=4 proteinuric mice per tissue, except for urine (control n=6, proteinuric n=3). For isotope-labeled metabolites, the sample size was n=6 control mice and n=5 proteinuric mice per tissue, except for urine (control n=6, proteinuric n=4). Pancreas and white adipose tissue were excluded from this analysis. For indophenol analyses, sample sizes varied slightly between tissues. For unlabeled indophenol, the sample size was n=6 control mice for most tissues, except for cortex, medulla, liver, jejunum, erythrocytes, and plasma (n=5). The number of proteinuric mice was n=4 for most tissues, except for cortex, medulla, lung, duodenum, and jejunum (n=3). For labeled indophenol, the sample size was n=6 control mice for most tissues, except for brain, colon, erythrocytes, lung, muscle, plasma, and spleen (n=5), and cortex and heart (n=4). The number of proteinuric mice was n=5 for most tissues, except for brain, colon, duodenum, erythrocytes, and ileum (n=4), and lung and muscle (n=3).

#### Analysis of isotope-labeled metabolites in isolated nephron segments.

The differential abundance of arginine-related isotope-labeled metabolites was calculated as the difference between the log_10_-transformed mean peak area in ^13^C-arginine-treated (n=8) and ^12^C-arginine-treated (n=8) segments. Statistical significance was assessed using a two-sided Mann–Whitney U test (P value < 0.05). Each mouse contributed two independent segment pools that were processed separately and therefore treated as independent samples.

#### Analysis of time-resolved isotope-labeled metabolites in isolated cortical tubules and glomeruli.

The time-resolved differential abundance of ^13^C-arginine–derived metabolites in cortical tubules and glomeruli was calculated as the difference between the log2-transformed mean heavy-to-unlabeled ratios in control and proteinuric mice. Statistical significance was assessed using a two-sided Mann–Whitney U test (P value < 0.05).

#### Differential abundance analysis of arginine-related metabolites in human serum samples.

The differential abundance of arginine-related metabolites in serum from nephrotic syndrome patients across albuminuria stages (A1 n=6, A2 n=6, A3 n=7) was calculated as log_2_ fold changes of mean peak areas between groups (log_2_(A3/A1), log_2_(A3/A2), and log_2_(A2/A1)). Statistical significance between groups was assessed using an unpaired two-sided Welch’s t-test (P value < 0.05). For longitudinal analyses, samples from the same patients were grouped into paired active–remission measurements in both the nephrotic syndrome cohort and an independent cohort of membranous nephropathy patients. The sample size was n=10 pairs for the nephrotic syndrome cohort and n=22 for the membranous nephropathy cohort. For each metabolite, paired log_2_ differences were calculated as log_2_ (remission/active). Statistical significance of paired changes was assessed using a one-sample t-test against zero.

#### Single-tubule protein detection analysis.

The number of detected proteins per tubule was calculated by counting unique proteins for each sample. To ensure robust detection, proteins were retained only if they were detected in more than 10 tubules within a given mouse. Summary statistics were computed across all tubules, including the mean ± SEM of detected proteins. In addition, per-mouse distributions were summarized using the number of tubules, mean, median, standard deviation, and range of detected proteins per tubule. These metrics were used to assess variability in proteome coverage across individual mice.

#### Principal component analysis of single-tubule proteomics data.

Principal component analysis (PCA) was performed to explore global variation in protein abundance across single-tubule samples obtained from two NPHS2^A286/+^ (Control_1, n=87 tubules; Control_2, n=95 tubules) and two NPHS2^A286V/A286V^ (Proteinuric_1, n=61 tubules; Proteinuric_2, n=57 tubules) mice. Missing protein abundance values were imputed only for the PCA analysis using k-nearest neighbors’ imputation (k=3) implemented in the KNNImputer function from the *scikit-learn* Python library^72^. Following imputation, protein abundance values were standardized using z-score scaling (mean = 0, variance = 1). PCA was performed using the PCA function from scikit-learn, and the first two principal components were used to visualize sample relationships.

#### Kaplan–Meier survival analysis.

Survival was defined as time to endpoint due to severe weight loss. Only weight loss–related deaths were considered events, and all other cases were censored. Kaplan–Meier curves were generated per genotype, and differences were assessed using global and pairwise log-rank tests with Benjamini–Hochberg correction. Summary survival statistics were calculated for each group.

#### Correlation analysis of albumin and arginine-/TCA cycle-related proteins in single-tubule proteomics data.

Protein abundance correlations were analyzed in the same dataset using a predefined panel of proteins involved in arginine metabolism and the TCA cycle. Data was analyzed separately for each mouse. Prior to analysis, the dataset was filtered to include only proteins detected in at least ten tubules per mouse. Albumin (Alb) was used as the reference protein, and pairwise Spearman rank correlations (ρ) between albumin abundance and the abundance of each selected protein were calculated across tubules of each mouse using pairwise complete observations. Correlations were computed only when sufficient overlapping observations were available^73–75^. Representative scatter plots of albumin versus Aldh18a1, Cps1, and Prodh were generated for individual mice, showing log_2_-transformed intensities across tubules together with Spearman correlation coefficients.

#### Longitudinal analysis of urinary albumin-to-creatinine ratio (uACR).

Urinary albumin-to-creatinine ratio (uACR) measurements were analyzed longitudinally in proteinuric mice, receiving either a control or a high aspartate/asparagine diet. Comparisons between proteinuric mice receiving the two diets were performed separately at each time point using a two-sided Mann–Whitney U test.

#### Urinary ammonium, pH and ammonium-pH index (API) analysis.

Changes in urinary NH_4_^+^, pH, and the ammonium–pH index across age were evaluated using mixed-effects models for repeated measures in StataBE 17.0 for Mac. The models included age, genotype, and the age-by-genotype interaction as fixed effects, with sex specified as a covariate. To account for within-mouse correlation, mouse ID was modeled as a random intercept, and age was additionally modeled as a random slope. To capture potential nonlinear relationships between age and the outcomes, age was parameterized using a restricted cubic spline with four knots.

#### Amino acid–resolved enrichment and labeling analyses of kidney proteome.

A final protein-level dataset was generated by merging two quantitative proteomics datasets. The first dataset comprised log_2_ fold changes from proteinuric mice fed an aspartate/asparagine diet versus control diet, while the second included channel-based comparisons (Pro6/unlabeled) between proteinuric and control mice under an arginine diet. Data were summarized at the gene level, representative UniProt identifiers were assigned, and amino acid composition was calculated from UniProt sequences. Only proteins with complete quantitative and sequence-derived data were retained. Proteins were ranked by amino acid composition, and the top 50–500 proteins per amino acid were analyzed to assess enrichment-dependent effects. Mean sign-inverted log_2_ fold changes (proteinuric vs control) and standard errors were visualized across thresholds, and their relationship to channel-specific differences was examined using scatter plots. For proline, the top 50 proteins were compared to all others using Welch’s t-test and Kolmogorov–Smirnov test, with distributions visualized using overlaid density-normalized histograms and mean indicators.

#### Data availability.

The mass spectrometry proteomics data have been deposited to the ProteomeXchange Consortium (http://proteomecentral.proteomexchange.org) via the PRIDE partner repository^42^. The raw data can be accessed for review through PRIDE/ProteomeXxhange^42^ via https://www.ebi.ac.uk/pride/login and the following accessions/reviewer tokens.

**Table:** 

Accession	Reviewer token	Comment
PXD075833	gLEy3KTQD0ee	Data-dependent Acquisition and OPEN search using Fragpipe (Fig. 1)
PXD075940	XCr8cklebrJz	Multi-organ proteomic analysis using isotope labeled and control mice using DIA (Fig. 1, 5)
PXD072523	tgVQ9VCrTfJn	Single tubule proteomics dataset (Fig. 3)
PXD075860	ohT9k24uMzGD	Aspartate/Asparagine diet (Fig. 6)

The mass spectrometry metabolomics data have been deposited to the massIVE repository^43^, using the following identifiers. They can be accessed through the following links.

**Table:** 

Accession number	doi	Link before publication	Comment
MSV000101202	doi:10.25345/C5BN9XH0G	ftp://MSV000101202@massive-ftp.ucsd.edu	Arginine Diet-Arginine Method (Fig. 2B, C; Fig. 5G)
MSV000101204	doi:10.25345/C5348GW3R	ftp://MSV000101204@massive-ftp.ucsd.edu	Arginine Diet−TCA cycle Method (Fig. 2C; Fig. 5G)
MSV000101205	doi:10.25345/C5ZC7S785	ftp://MSV000101205@massive-ftp.ucsd.edu	Nephron segments - Arginine method (Fig. 3B)
MSV000101206	doi:10.25345/C5TQ5RT6V	ftp://MSV000101206@massive-ftp.ucsd.edu	Tubules-Arginine method (Fig. 3D)
MSV000101207	doi:10.25345/C5PZ52130	ftp://MSV000101207@massive-ftp.ucsd.edu	Glomeruli-Arginine method (Fig. 3D)
MSV000101211	doi:10.25345/C55X25S6D	ftp://MSV000101211@massive-ftp.ucsd.edu	Arginine Diet-Indophenol Method- part1 (Fig. 5E, F)
MSV000101213	doi:10.25345/C5XG9FR0C	ftp://MSV000101213@massive-ftp.ucsd.edu	Arginine Diet-Indophenol Method- part2 (Fig. 5E, F)
MSV000101218	doi:10.25345/C58P5VQ0J	ftp://MSV000101218@massive-ftp.ucsd.edu	Arginine Diet-Indophenol Method- part 3 (Fig. 5E, F)

Due to privacy and GDPR regulations and requirements^76^, spectral data from humans cannot be shared openly but aggregated data are fully presented in this manuscript. Furthermore, the underlying raw data is available from the corresponding author and research institution upon signing a data transfer agreement.

## Supplementary Material

1

## Figures and Tables

**Fig. 7.**
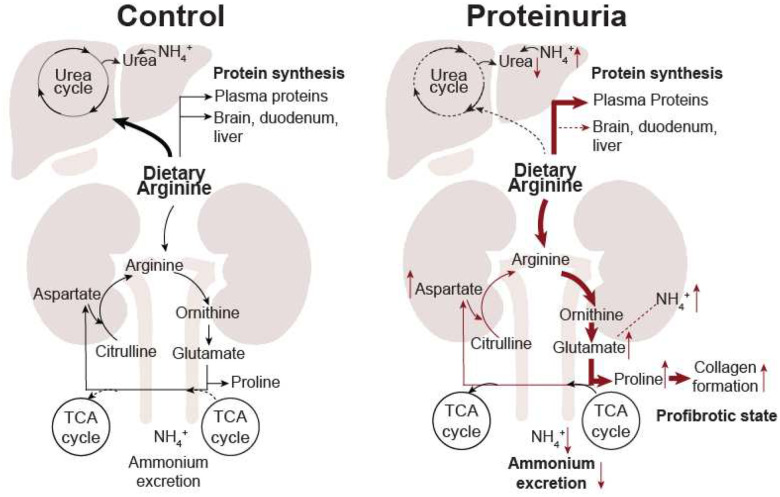
Conclusion: Dietary isotope labeling enables simultaneous tracking of proteomic and metabolomic nutrient fates across organs, revealing how nephrotic range proteinuric kidney disease reshapes systemic arginine metabolism. Under steady-state conditions, arginine is predominantly directed toward hepatic ureagenesis, supporting high urea-cycle activity for ammonium detoxification, while albumin synthesis remains relatively low. In proteinuria, this balance is disrupted: urea-cycle function is impaired, urea synthesis declines, and arginine is increasingly diverted toward plasma albumin production. This compensatory albumin synthesis occurs at the expense of protein turnover in extrahepatic tissues, including the intestine and brain. At the same time, recycled arginine is increasingly repurposed within the kidney, promoting ornithine, glutamate, and proline formation while increasing aspartate. Enhanced proline synthesis and collagen incorporation indicate activation of a profibrotic renal program. Together, these shifts link nephrotic-range proteinuria to increased extrahepatic arginine catabolism, prioritization of albumin replacement, ammonium retention and renal diversion of arginine toward proline-dependent matrix remodeling. The accompanying perturbation in aspartate availability provides a metabolic connection between altered arginine handling, a profibrotic state, and acid retention in proteinuric kidney disease.

## Key points:

- Long-term dietary arginine isotope tracing maps proteinuria-associated amino-acid fate across metabolites and proteins in multiple organs.
- Proteinuria does not induce a generalized increase in protein synthesis, but redirects dietary arginine utilization away from hepatic ureagenic labeling toward renal arginine-derived metabolism.
- During proteinuria, the proximal tubule becomes a pre-fibrotic metabolic sink that converts arginine-derived carbon and nitrogen toward proline synthesis and collagen incorporation.
- Altered ammonium handling and aspartate/asparagine-linked metabolism connect proteinuria to acid retention, disrupted nitrogen disposal, and profibrotic remodeling.
