# Supplementary material for: Diversion of Arginine’s dietary metabolic fate in proteinuric kidney disease
Source: Res Sq. 2026 Jun 17:rs.3.rs-9927381. Preprint. [Version 1] doi: 10.21203/rs.3.rs-9927381/v1 (PMC13308378; doi:10.21203/rs.3.rs-9927381/v1)
Supplement: 1 [file NIHPPRS9927381V1-supplement-1.pdf]

Suppl. Figures

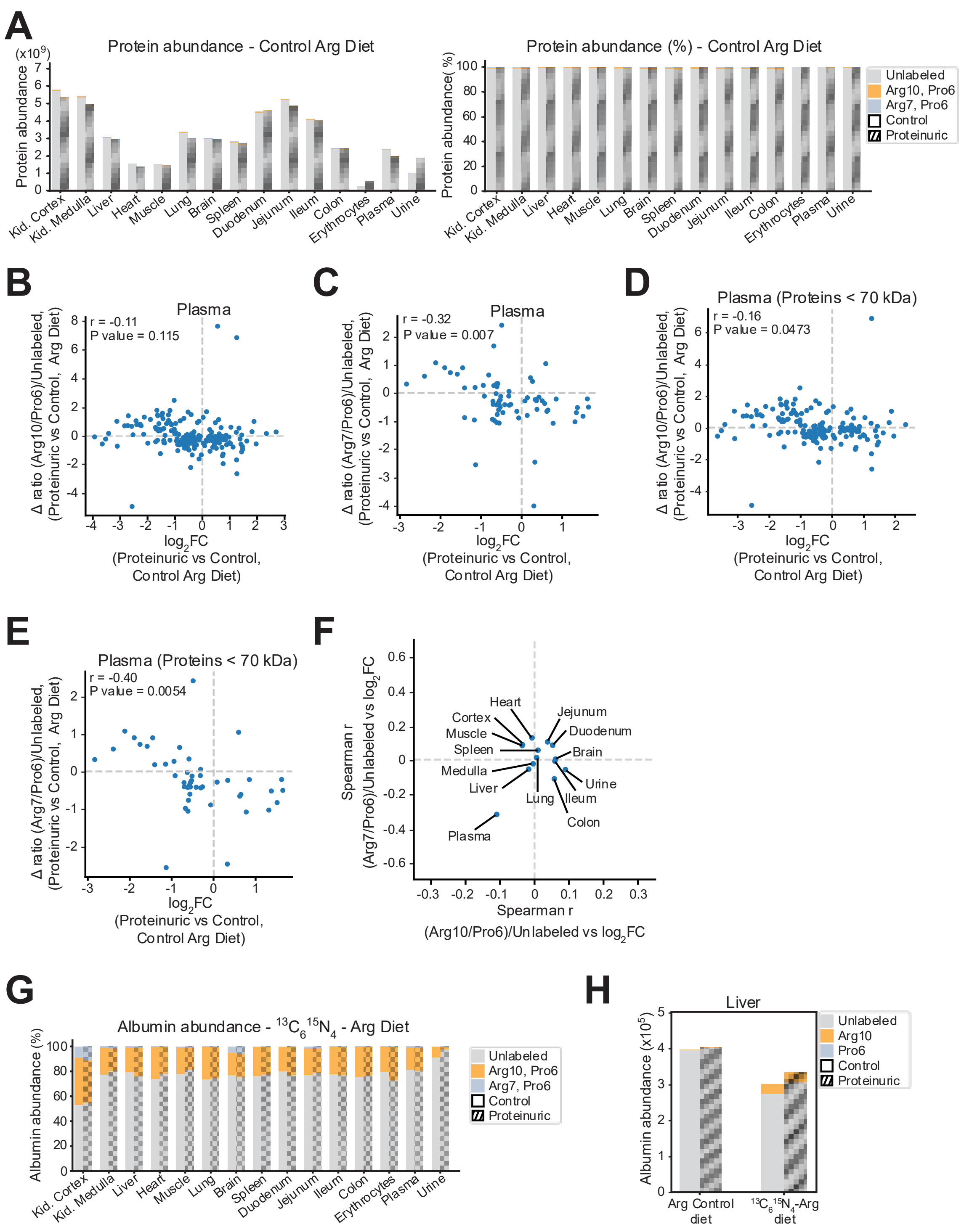

Suppl. Figure 1. Protein synthesis is buffered in proteinuria.

Supplementary Figure 1. **Assessment of arginine contribution to global protein synthesis in proteinuria. A.** System-wide absolute (left) and percent (right) protein abundance after incorporation of Arg10/Pro6 and Arg7/Pro6 in mice fed the control arginine diet. **B.** Spearman correlation between protein abundance changes and labeling enrichment of Arg7/Pro6. Each point represents a protein. **C.** Spearman correlation between protein abundance changes and labeling enrichment of Arg10/Pro6. Each point represents a protein. **D-E.** Same as B-C, for proteins with a molecular mass of less than 70 kDa. **F.** Tissue-specific Spearman correlations between labeling ratios and protein $\log_2$ fold change. Points represent tissues. **G.** System-wide percent albumin abundance after incorporation of Arg10/Pro6 and Arg7/Pro6 in mice fed the $^{13}C_6{}^{15}N_4$-arginine diet. **H.** Absolute liver albumin abundance in mice fed the $^{13}C_6{}^{15}N_4$-arginine diet after incorporation of Arg10 and Pro6.

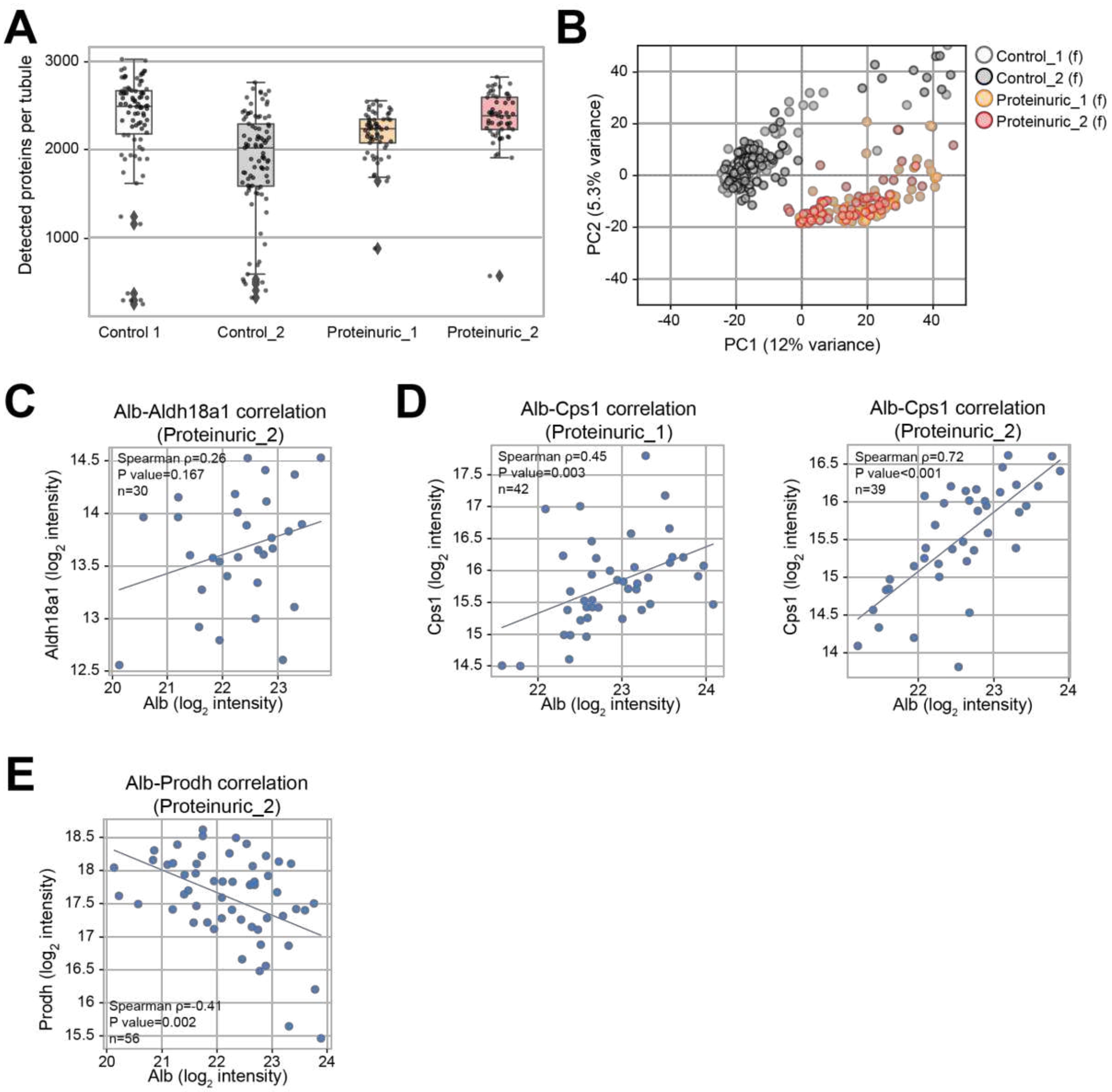

Supplementary Figure 2. Single-tubule proteomics profiling in control and proteinuric mice. A. Number of detected proteins per tubule in individual control and proteinuric mice**.** Each dot represents a tubule. Data are shown as boxplots (median and interquartile range), with whiskers indicating the spread of the data. **B.** Principal component analysis of single-tubule proteomics profiles. Missing values were imputed using k-nearest neighbors (k=3), and protein abundances were standardized prior to PCA. Each point represents a single tubule. **C-E.** Spearman correlation between Alb and Aldh18a1, Cps1, and Prodh log_2 intensities, respectively. Each point represents a tubule.

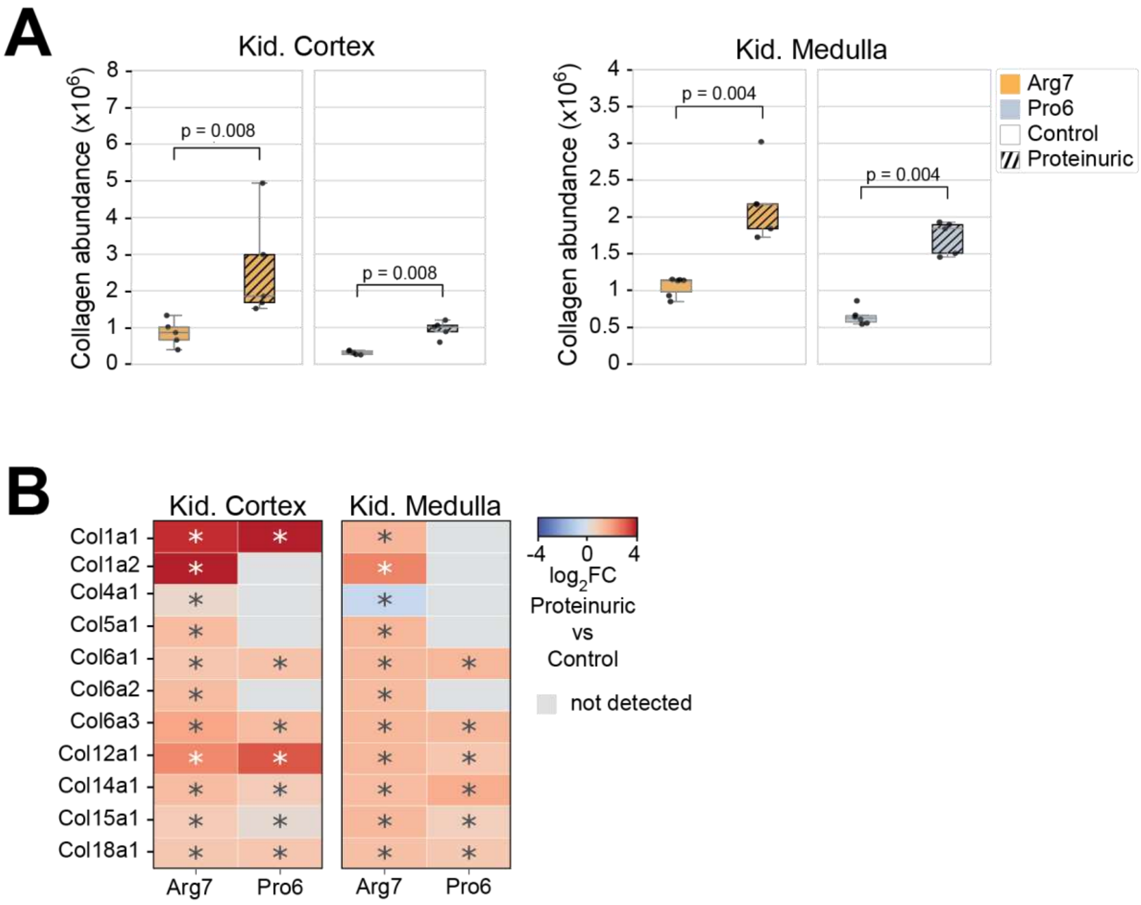

Supplementary Figure 3. Abundance of Arg7- and Pro6-enriched collagens in kidney cortex and medulla of proteinuric animals. A. Per-mouse summed collagen intensities reflecting incorporation of labeled amino acids (Arg7, Pro6) in control and proteinuric mice. Each dot represents one mouse. Boxes indicate the median and interquartile range (IQR), and whiskers extend to 1.5 × IQR. P values were calculated using a two-sided Mann–Whitney U test. **B.** Differential abundance of collagen proteins enriched in labeled amino acids (Arg7, Pro6) in kidney cortex and medulla between control and proteinuric mice.

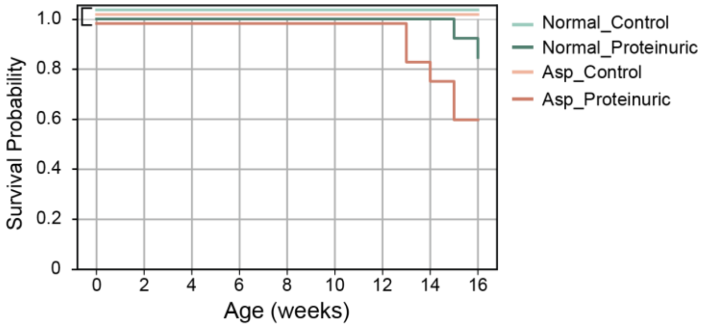

Suppl. Figure 4. Survival data from the aspartate/asparagine supplementation study. Kaplan–Meier survival curves showing survival probability over time. Statistics are included in Suppl. Table 28, demonstrating increased mortality with Aspartate/Asparagine diet on proteinuria.

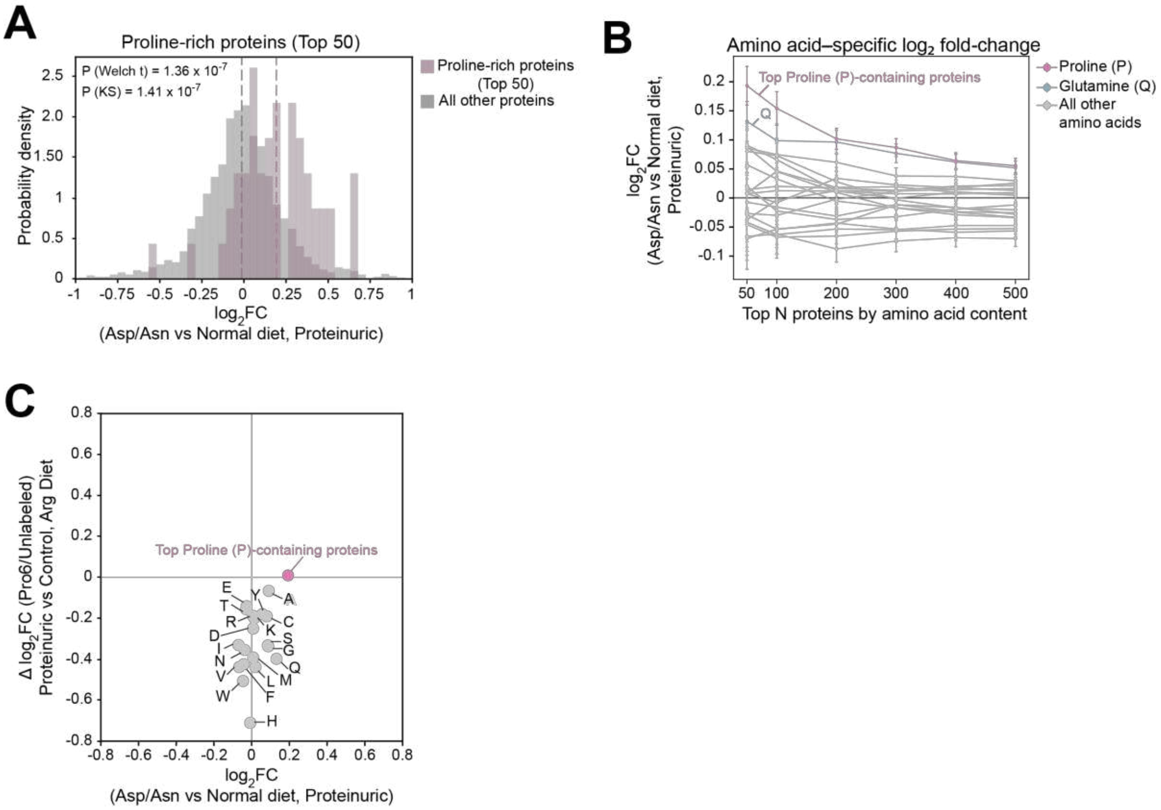

Suppl. Figure 5. High aspartate/asparagine diet increases proline-containing proteins. A. Distribution of $\log_2$ fold changes (Asp/Asn enriched vs normal det) for the top 50 proline-rich proteins compared with all remaining proteins in proteinuric mice. Data are shown as probability density. Dashed lines indicate group means. Statistical significance was assessed using Welch's t-test (P value = 5.3×10^{-6}) and the Kolmogorov–Smirnov test (P value = 3.1×10^{-6}). **B.** Amino acid–specific trajectories of average $\log_2$ fold-change (Asp/Asn vs normal diet, proteinuric mice) across increasing top N protein subsets (50–500), ranked by amino acid content. Values are mean ± SEM. **C.** Amino acid–specific comparison of average $\log_2$ fold-change after Asp/Asn diet in proteinuric mice and $\Delta \log_2$ FC from Pro6 incorporation between proteinuric and control mice, based on the top 50 proteins ranked by amino acid content. Each point represents one amino acid.

Supplementary tables and source data.

Supplementary Table 1. System-wide differential expression of proteins between control and proteinuric mice by multi-organ proteomics.

Supplementary Table 2. System-wide KEGG pathway enrichment analysis between control and proteinuric mice from multi-organ proteomic data.

Supplementary Table 3. Amino acid–specific mass shifts identified by open search using FragPipe.

Supplementary Table 4. Protein quantification across labeling channels obtained from Spectronaut analysis.

Supplementary Table 5. Plasma proteins used for correlation between log_2 fold-change (Normal diet, proteinuric vs control) and labeling ratios.

Supplementary Table 6. Plasma proteins below 70kDa used for correlation between log_2 fold-change (Normal diet, proteinuric vs control) and labeling ratios.

Supplementary Table 7. Tissue-specific Spearman correlations between protein abundance changes and isotope labeling enrichment (Arg10/Pro6 and Arg7/Pro6).

Supplementary Table 8. Albumin quantification across labeling channels (Arg10/Pro6 and Arg7/Pro6) from Spectronaut analysis.

Supplementary Table 9. Albumin quantification in the liver across labeling channels (Arg10 and Pro6) from Spectronaut analysis.

Supplementary Table 10. System-wide differential abundance of arginine-related metabolites between control and proteinuric mice.

Supplementary Table 11. System-wide differential abundance of isotope-labeled arginine-related metabolites between control and proteinuric mice.

Supplementary Table 12. Differential isotopic enrichment of arginine-related metabolites between $^{13}C_6$-Arginine and $^{12}C_6$-Arginine treatments across kidney segments.

Supplementary Table 13. Time-resolved arginine-derived isotopic enrichment in tubules and glomeruli between control and proteinuric mice.

Supplementary Table 14. Number of detected proteins per tubule in control and proteinuric mice.

Supplementary Table 15. Single-tubule protein abundance matrix used for PCA analyses.

Supplementary Table 16. Spearman correlation of arginine- and TCA cycle–related proteins with albumin in single tubules.

Supplementary Table 17. Collagen quantification across labeling channels (Arg10/Pro6, Arg7/Pro6) from Spectronaut analysis.

Supplementary Table 18. Collagen quantification across labeling channels (Arg10, Pro6) from Spectronaut analysis.

Supplementary Table 19. Collagen quantification across labeling channels (Arg7, Pro6) from Spectronaut analysis.

Supplementary Table 20. Differential expression of collagens enriched with arginine-derived labeled amino acids (Arg10, Pro6) and (Arg7, Pro6) in kidney cortex and medulla between control and proteinuric mice.

Supplementary Table 21. Ammonium and pH levels in control and proteinuric mice.

Supplementary Table 22. System-wide differential abundance of unlabeled and isotope-labeled indophenol between control and proteinuric mice.

Supplementary Table 23. System-wide differential abundance of ^{15}N-labeled arginine-related metabolites between control and proteinuric mice.

Supplementary Table 24. System-wide differential expression of arginine-related proteins between control and proteinuric mice.

Supplementary Table 25. Differential abundance of arginine-related metabolites in patient serum between albuminuria stages.

Supplementary Table 26. Differential abundance of arginine-related metabolites in serum from patients with nephrotic syndrome between active disease and remission states.

Supplementary Table 27. Differential abundance of arginine-related metabolites in serum from membranous nephropathy patients between active disease and remission states.

Supplementary Table 28. Urinary albumin to creatinine ratio in proteinuric mice fed a normal or high aspartate/asparagine diet.

Supplementary Table 29. Kaplan–Meier survival analysis of control and proteinuric mice fed a control and a high aspartate/asparagine diet.

Supplementary Table 30. Ammonium-pH index (API) in proteinuric mice fed a normal or high aspartate/asparagine diet.

Supplementary Table 31. Differential expression of collagens in proteinuric mice fed a normal or high aspartate/asparagine diet.

Supplementary Table 32. Differential expression of arginine-related proteins in proteinuric mice fed a normal or high aspartate/asparagine diet.

Supplementary Table 33. Protein-level dataset and statistical comparison of top amino acid–enriched proteins versus remaining proteins, with proline-focused analysis and sequence composition.

Supplementary Table 34. Mean ± SEM of $\log_2$ fold-change (proteinuric vs control, Asp/Asn diet) and labeling ($\log_2$FC Pro6/Unlabeled proteinuric versus control in Arg diet) across amino acid–ranked top N protein subsets (50–500).

Supplementary Table 35. MRM transitions and parameters for QQQ LC–MS targeted metabolomics analysis.

Supplementary Files

This is a list of supplementary files associated with this preprint. Click to download.

- 20260428SupplementaryTable7.xlsx
- 20260325SupplementaryTable3.xlsx
- 20260428SupplementaryTable12.xlsx
- 20260428SupplementaryTable9.xlsx
- 20260428SupplementaryTable19.xlsx
- 20260428SupplementaryTable17.xlsx
- 20260428SupplementaryTable13.xlsx
- 20260428SupplementaryTable31.xlsx
- 20260428SupplementaryTable23.xlsx
- 20260428SupplementaryTable27.xlsx
- 20260428SupplementaryTable18.xlsx
- 20260428SupplementaryTable21.xlsx
- 20260428SupplementaryTable8.xlsx
- 20260428SupplementaryTable28.xlsx
- 20260430SupplementaryTable22.xlsx
- 20260428SupplementaryTable14.xlsx
- 20260428SupplementaryTable16.xlsx
- 20260428SupplementaryTable10.xlsx
- 20260423SupplementaryTable6.xlsx
- 20260309SupplementaryTable2.xlsx
- 20260428SupplementaryTable24.xlsx
- 20260428SupplementaryTable5.xlsx
- 20260428SupplementaryTable26.xlsx
- 20260428SupplementaryTable25.xlsx
- 20260428SupplementaryTable29.xlsx
- 20260428SupplementaryTable11.xlsx
- 20260428SupplementaryTable20.xlsx
- 20260309SupplementaryTable1.xlsx
- 20260428SupplementaryTable30.xlsx
- 20260428SupplementaryTable15.xlsx
- 20260423SupplementaryTable4reduced.xlsx
